# Reward processing deficits: weakened self-reward association in individuals with methamphetamine addiction undergoing abstinence

**DOI:** 10.3389/fpsyg.2025.1567735

**Published:** 2025-09-01

**Authors:** Xie Yanghui, Fan Chong, Li Qianyu

**Affiliations:** ^1^Key Laboratory of Cognition and Human Behavior in Hunan Province, School of Education Science, Hunan Normal University, Changsha, Hunan, China; ^2^College of Humanities and Arts, Hunan International Economics University, Changsha, Hunan, China; ^3^College of Management Science and Engineering, Hunan University of Technology and Business, Changsha, Hunan, China; ^4^Public Mental Health Prevention Office, The Ninth Hospital of Changsha, Changsha, Hunan, China

**Keywords:** self-processing, reward processing, addiction, methamphetamine, Go/No-Go paradigm

## Abstract

This research primarily investigates whether both reward processing and self-processing are aberrant in individuals with methamphetamine use disorder. It also explores whether initiating self-referential processing modulates reward processing abilities in this population, and how this modulation differs from that observed in healthy controls. Experiment 1 employed a two-factor mixed experimental design to compare the performance of addiction groups with varying withdrawal durations (all participants in the addiction groups were methamphetamine users) against healthy control groups in a probabilistic reward learning task. The results indicated that the healthy control group performed better than the addiction group in learning characters associated with high-probability, high-reward outcomes. While the long-term abstinence group outperformed the short-term abstinence group, these differences were not statistically significant. Therefore, the addiction group subjects in Experiments 2 and 3, the addiction group consisted of methamphetamine users with a uniform withdrawal duration of no more than 12 months. Experiment 2 utilized a two-factor mixed design to explore whether self-processing is abnormal in addicted individuals. The results showed that the addiction group had a significantly lower accuracy rate for self-referential characters compared to the healthy control group, while their accuracy for characters associated with acquaintances was significantly higher than that of the healthy control group. Experiment 3 also employed a two-factor mixed design to examine the moderating effect of self-processing on reward learning. The findings revealed that when high-probability reward characters were linked to self-relevance, learning efficiency was superior to that of characters linked to acquaintances. However, this moderating effect was weaker in the addiction group compared to the healthy control group. These results suggest that substance addiction not only impairs individuals' reward processing abilities but also reduces their sensitivity to self-referential information. Furthermore, the enhancing effect of self-processing on reward learning is significantly diminished in addicted populations, providing new insights into the cognitive mechanisms underlying addiction.

## 1 Introduction

Substance use disorder manifests as a constellation of behavioral characteristics, including persistent efforts to obtain the substance, compulsive usage patterns, and continued use despite experiencing negative consequences (Roehr, 2013). Given the high prevalence of methamphetamine use disorder in China (The 2019 China Drug Report indicated that, of the 2.148 million existing drug users, 1.186 million abused synthetic drugs, primarily methamphetamine, representing 55.2% of all drug users, making methamphetamine the most widely abused drug in China; The 2021 China Drug Report stated that by the end of 2021, there were 1.486 million existing drug users in China. While drug abuse has shown some improvement, a significant number of new drug users continue to emerge.), this study specifically focuses on the cognitive mechanisms underlying methamphetamine addiction, a prevalent form of substance use disorder closely associated with cognitive impairment. The academic community has extensively studied the behavioral abnormalities and underlying causes of drug addiction, which is considered a chronic brain disease characterized by aberrant reward processing (Volkow and Morales, 2015). This leads to heightened responses to drugs and drug-associated cues (Hayes et al., 2020), while natural rewards and related stimuli elicit diminished responses (Garfield et al., 2014; Koob and Volkow, 2016). Research indicates that these abnormalities in reward processing are a primary factor contributing to addiction (Adinoff, 2004). Specifically, Koob and Volkow (2010) posits that addiction is linked to the rewarding effects of addictive substances or behaviors, identifying the mesocorticolimbic dopamine system as a crucial neural circuit in addiction development. Moreover, the impact of addictive drugs on the nervous system further disrupts natural reward responses (Keiflin and Janak, 2015). Gardner (2011) and Hayes et al. (2020) elaborate on the intrinsic neural mechanisms of drug addiction, suggesting that drug use increases dopamine release in the mesocorticolimbic system, inducing feelings of euphoria and pleasure and this reinforcing effect contributes to the development of addictive behaviors. Additionally, Ivanov et al. (2019) provide further evidence that children exhibiting aberrant reward processing are more likely to develop substance abuse later in life, and this processing can be partially restored through abstinence (Yang et al., 2015; Ceceli et al., 2022; Balodis et al., 2016).

Beyond aberrant reward processing, a growing body of research suggests that self-processing may also be dysfunctional in individuals with addiction. While current research primarily suggests the possibility of aberrant self-processing in addicted individuals from a neurobiological perspective—for example, Nocjar and Panksepp (2007) identified the ventromedial prefrontal cortex (VMPFC), a brain region crucial for self-processing and implicated in the development of drug addiction (Adinoff, 2004), as vulnerable to functional impairment due to prolonged drug exposure (Nocjar and Panksepp, 2007; Jacobs-Brichford et al., 2019; Yan et al., 2023)—we acknowledge that there is a lack of direct behavioral studies investigating self-processing in addiction. Indeed, to our knowledge, no prior study has investigated self-processing in addiction in animals or humans using behavioral measures. This gap in the literature highlights the novelty and significance of our study, which aims to explore the behavioral manifestations of aberrant self-processing in individuals with methamphetamine addiction and its interaction with reward processing.

Given that both self-processing and reward processing may be aberrant in individuals with addiction, and that both may contribute to addictive behaviors (Volkow and Morales, 2015), understanding the relationship between these two processes is crucial. Northoff and Hayes (2011) proposed three models to describe this relationship: (1) the integrated model, suggesting shared underlying mechanisms; (2) the separate model, proposing independence; and (3) the parallel processing model, which suggests that different aspects of self-referential processing occur in parallel with reward processing, yet interact. While current research leans toward the parallel processing model, the precise nature of this interaction remains unclear. Madan et al. (2010) suggested that self-relevant stimuli and their processing play a role in various psychological processes in ways similar to, or interacting with, reward processing (Madan et al., 2010; Yi-Beng et al., 2018). Kwak et al. (2014) found that individuals learned reward rules more quickly in self-relevant tasks, further supporting a link between these processes. Although Northoff and Hayes's (2011) model provides a valuable theoretical framework, empirical studies specifically examining the impact of self-processing on reward processing in the context of addiction remain limited. Previous research has primarily focused on either reward processing deficits or self-related cognitive impairments in addiction, often without directly linking the two. Therefore, this study aims to extend this line of research by directly investigating the interaction between self-processing and reward processing in abstinent methamphetamine addicts, exploring how and why reward processing is affected.

Neuroimaging studies have revealed overlapping neural networks engaged in both self-referential processing and reward processing (Kelley and Berridge, 2002; Northoff et al., 2006). Within the framework of valuation theories, particularly concerning the VMPFC, stimuli or representations associated with the self are hypothesized to undergo preferential valuation, thereby acquiring heightened salience and motivational relevance (D'Argembeau, 2013; Tacikowski et al., 2013). Reward processing, characterized by the experience of positive affect, the instigation of approach-oriented behaviors, and the facilitation of associative learning, critically depends on the capacity to assign subjective value to rewarding stimuli or events. Consequently, self-referential processing, via its inherent value-assignment mechanisms, may exert a foundational or modulatory influence on reward processing. However, studies examining individuals with addiction reveal a disruption in this relationship. While healthy controls typically activate brain regions associated with both self-processing and reward processing, individuals with addiction often exhibit reduced activation in reward-related brain regions during self-referential processing compared to healthy controls (De Greck et al., 2010; Zhu and Zhan, 2019). This suggests the presence of aberrant self-referential processing in individuals with addiction, potentially indicative of deficits in reward valuation. Thus, dysfunctional self-referential processing may contribute to, or exacerbate, aberrant reward processing commonly observed in addiction. Despite these theoretical and empirical links, research directly examining the precise nature of the relationship between self-referential processing and reward processing, and the specific role of self-referential processing in modulating reward-related behavior in individuals with addiction, remains limited.

First, previous research on Homo sapiens has focused on whether the brain mechanisms of reward processing in addicted Homo sapiens groups are abnormal, and whether the reward processing outcomes in behavioral experiments are similarly abnormal in addicted Homo sapiens groups. Can this abnormality be restored through abstinence? Second, the relationship between the self and reward is closely linked at both functional and neural levels. Studies show that the self-reference effect in healthy Homo sapiens groups enhances memory performance for self-related materials. However, due to long-term drug exposure and damage to reward-related brain regions, do addicted Homo sapiens groups still maintain this self-reference effect? Finally, self-processing and reward processing are functionally similar and exhibit overlapping neural activation (Zhan et al., 2016), but their intrinsic relationship remains unclear. Research indicates that self-processing can enhance reward processing, but is this regulatory effect strengthened or weakened in addicts? After damaging the relevant brain regions, which model do the self and reward processing of addicts follow? To address these questions, we conducted three experiments to investigate reward processing, self-processing, and the interaction between self and reward in individuals with MA addiction undergoing abstinence.

Experiment 1 utilizes a Go/No-Go paradigm (Frank et al., 2004; Schevernels et al., 2016. Wypych et al., 2019; Maruo and Masaki, 2024) to investigate reward processing in individuals with addiction, validating whether reward processing is indeed aberrant and whether abstinence reverses this abnormality. We hypothesize that methamphetamine addicts will exhibit significantly lower accuracy rates than the healthy control group when selecting high-probability/high-reward stimuli in the Go/No-Go task. Within the addict population, long-term abstainers will demonstrate higher accuracy rates than short-term abstainers when choosing high-probability/high-reward stimuli. Experiment 2 employs a self-referential paradigm (Sui et al., 2012) to explore whether self-processing related to reward processing is also aberrant in individuals with addiction. We hypothesize that the accuracy rates of both the addiction group and the healthy control group in self-judgment trials will be significantly higher than those in familiar Homo sapiens judgment trials. The accuracy rate of the healthy control group in self-judgment trials will be significantly higher than that of the addiction group. Experiment 3 will investigate whether self-processing exerts a modulating (enhancing or diminishing)effect on reward processing in individuals with addiction, and how this differs from healthy controls. We hypothesize that, regardless of whether in the addiction group or the healthy control group, when high-probability reward stimuli are associated with the self (compared to when associated with a familiar other homo sapiens), participants will exhibit higher response accuracy. Compared to the healthy control group, the addiction group will show a significantly diminished increase in response accuracy when facing self-related high-probability reward stimuli.

These experiments aim to elucidate the relationship between self-processing and reward processing and contribute to a better understanding of the aberrant behaviors and underlying mechanisms of addiction.

## 2 Experiment 1: aberrant reward processing in individuals with methamphetamine addiction

### 2.1 Methods

#### 2.1.1 Experimental design

A 2 (reward level: low, high) × 3 (group: short-term abstinence, long-term abstinence, healthy control) mixed-design was employed to investigate the effect of reward level on decision-making behavior in individuals with methamphetamine addiction undergoing abstinence. Reward level was a within-subject variable, while group was a between-subject variable. Yi ethnic vowel characters, which to our knowledge have not been used in previous studies as reward stimuli, were used as reward stimuli to control for potential cultural and linguistic influences. The dependent variable was the accuracy rate in choosing high-probability characters associated with high rewards.

#### 2.1.2 Participants

This study received ethical approval (Review Number: 671). A priori power analysis using G^*^Power 3.1.9 with an effect size *f* = 0.25, significance level α = 0.05, and power (1–β) = 0.95, indicated a minimum of 66 participants were required. A total of 76 participants meeting DSM-5 criteria for methamphetamine addiction were divided into two groups: a short-term abstinence group (3–12 weeks of abstinence) and a long-term abstinence group (6–12 months of abstinence). These criteria were chosen to reflect clinically relevant stages of recovery, with the short-term group representing the acute withdrawal phase and the long-term group representing a more sustained period of abstinence. While a definitive standard for these specific durations is lacking, similar timeframes have been used in previous research to differentiate between early and prolonged abstinence in stimulant addiction (Fernandez et al., 2020; Scott et al., 2011; Kolb and Himmelsbach, 1938). All participants were right-handed, had normal or corrected-to-normal vision, no history of alcohol abuse or dependence, no history of traumatic brain injury, neurological disorders, or psychiatric illnesses, and had not taken any medication potentially affecting neurological function in the week prior to the experiment. Participants with addiction were recruited from detoxification centers in Beijing and Changsha. Participants were stringently screened to ensure they were primarily methamphetamine users, with no history of other substance use disorders. The Structured Clinical Interview for DSM-5 (SCID) was used to exclude participants with neurological or psychiatric disorders. All participants provided informed consent after a thorough explanation of the experimental procedures. Participants received compensation for their participation (addiction groups received prizes due to center regulations). Demographic data is presented in Table 1.

**Table 1:** Demographic variables by group (*N* = 100).

**Group**	***n***	**Mean age (standard error)**	**Gender ratio (Male:female)**	**Education level (standard error)**
Long-term abstinence	40	31.48 (0.926)	20:20	9.3 (0.547)
Short-term abstinence	36	31.75 (0.99)	21:15	10.11 (0.4)
Healthy control	24	29.21 (0.976)	18:6	9 (0.614)

To ensure the validity of the data, we performed a chi-square test to assess potential differences in gender distribution across the healthy control group, short-term abstinence group, and long-term abstinence group. The results indicated no statistically significant differences in gender distribution among the three groups (χ^2^_(2)_ = 3.886, *p* = 0.143).

#### 2.1.3 Materials

**Figure 1:**
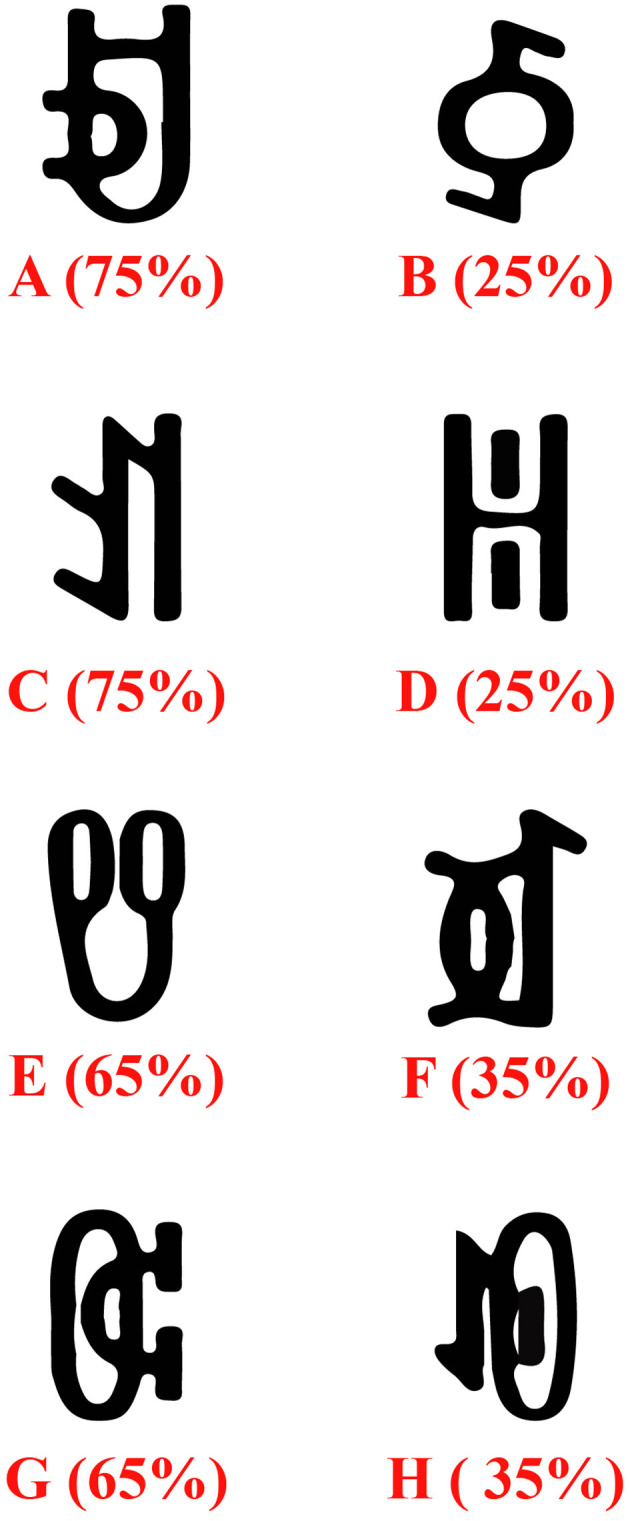
The experimental materials included eight Yi vowel characters as reward stimuli. The letters A, B, C, D, E, F, G, and H each represented one character, and the numbers in parentheses indicated the probability of obtaining a high-score reward.

Eight Yi ethnic vowel characters (A, B, C, D, E, F, G, H; see Figure 1) served as reward stimuli. To ensure stimulus validity, a 7-point scale was used to assess differences in arousal, familiarity, and complexity across the eight characters. Results showed no significant differences across these three dimensions (arousal: *M* = 1.87, *SD* = 0.12; familiarity: *M* = 0.82, *SD* = 0.07; complexity: *M* = 2.65, *SD* = 0.19). During the experiment, reward stimuli were presented in pairs. Each pair corresponded to either a high-probability high-reward or low-probability low-reward condition, and a low-probability high-reward or high-probability low-reward condition. The total probability of obtaining a high score for each pair remained constant at 1 (e.g., when characters A and B appeared together, A had a 75% probability of a high score, and B had a 25% probability). Participants learned to select the character with a higher probability of obtaining a high reward to maximize their final score.

#### 2.1.4 Procedure

##### 2.1.4.1 Experimental stages

The experiment, programmed and recorded using E-Prime 2.0 software, consisted of a learning phase and a testing phase. The learning phase randomly presented four character pairs, each representing different reward probabilities: one pair with a high probability of a 9-point reward, and another with a high probability of a 1-point reward. Participants selected characters by pressing “F” or “J,” with immediate feedback (“+9” or “+1”). The task was to identify the character pair with a high probability of a 9-point reward. Character pairs appeared randomly, with position and probability balanced across participants. The learning phase comprised four blocks of 120 trials each. Following the learning phase, a test phase assessed participants' understanding of the probabilities associated with high- and low-reward characters. The test included two types of pairings: (1) characters A or C paired with E, F, G, or H; and (2) characters B or D paired with E, F, G, or H. Participants chose A or C for high scores and E, F, G, or H to avoid the lowest score. Accuracy exceeding 0.5 in all conditions was required to proceed to the formal test. To test participants' learning and discrimination of high-probability high-reward characters (A, C) and high-probability low-reward characters (B, D), the test phase randomly presented A, C, B, and D paired with E, F, G, and H, with each pair presented six times. No feedback was given after each selection; only the final total score was displayed.

##### 2.1.4.2 Experimental operations

**Figure 2:**
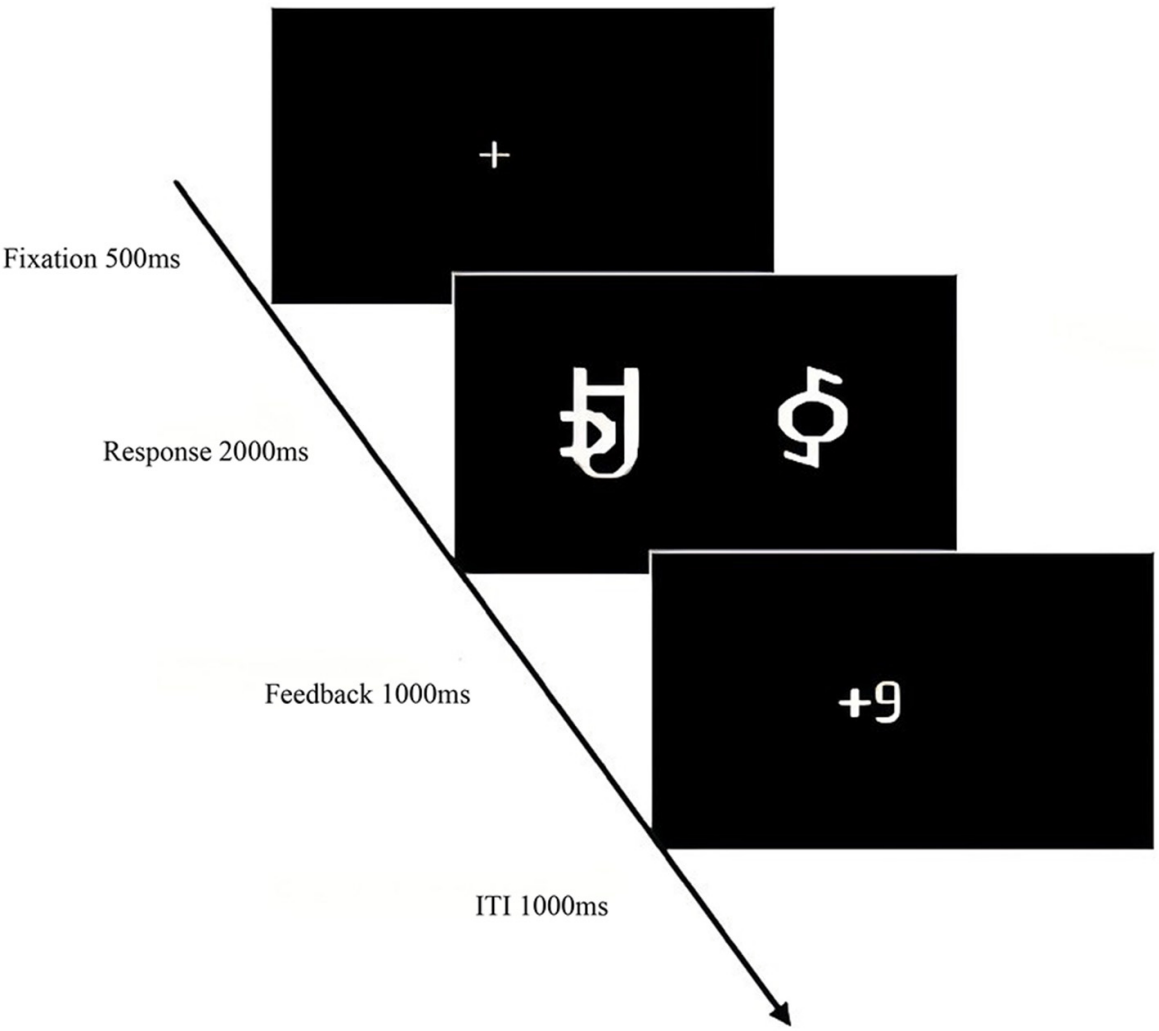
Experimental procedure for the reward processing task learning phase in Experiment 1.

**Figure 3:**
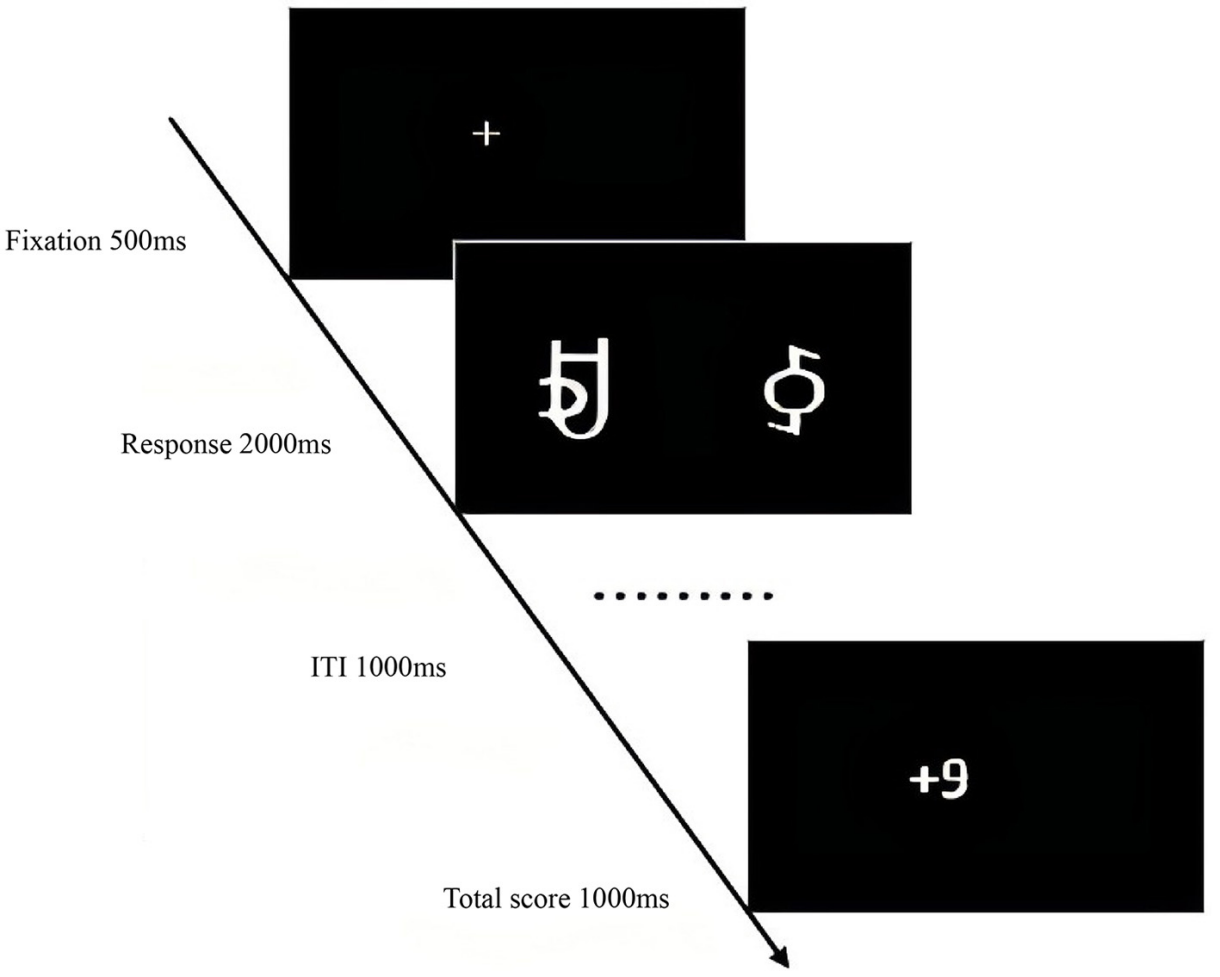
Experimental procedure for the reward processing task in Experiment 1.

The experiment began with a central fixation point “+” (500 ms), followed by the random presentation of reward stimuli pairs. Participants selected the left or right character within 2,000 ms by pressing “F” or “J.” The selected character's score was then displayed (1,000 ms). The learning phase comprised 480 trials with an inter-trial interval (ITI) of 1,000 ms. The testing phase began similarly with the fixation point “+” (500 ms), followed by the presentation of reward stimuli pairs. Participants selected the character pair with the higher probability of winning within 2,000 ms. A total of 16 pairs were presented six times each, with an ITI of 1,000 ms. The total experiment duration was ~50 min (Figures 2, 3).

#### 2.1.5 Data analysis

During the learning phase, participants' accuracy in selecting high-probability high-reward characters was analyzed using a one-way ANOVA, examining overall accuracy and accuracy within each block. A separate one-way ANOVA, with accuracy in each block as the dependent variable, was conducted to assess performance across learning stages.

In the testing phase, a repeated-measures ANOVA was conducted with two probability discriminations (high-probability high-reward A&C, low-probability high-reward B&D) and three participant groups (long-term abstinence, short-term abstinence, healthy controls).

### 2.2 Results

#### 2.2.1 Learning phase

All participants showed a tendency to select high-probability high-reward characters, with overall accuracy exceeding 0.5. However, significant group differences emerged in accuracy across different probability conditions: for the 65% probability condition [*F*_(2, 194)_ = 9.649, *p* < 0.001, $\eta_{p}^{2}$ = 0.294]; for the 75% probability condition [*F*_(2, 194)_ = 5.997, *p* < 0.05, $\eta_{p}^{2}$ = 0.253]; and for overall accuracy across both probability conditions [*F*_(2, 194)_ = 8.057, *p* < 0.001, $\eta_{p}^{2}$ = 0.346]. *Post-hoc* comparisons revealed that the healthy control group exhibited significantly higher accuracy than both the long-term and short-term abstinence groups (*F* > 5.63, *p* < 0.05), with no significant difference between the long-term and short-term abstinence groups (*p* > 0.05).

To further investigate performance across learning stages, a one-way ANOVA was conducted using accuracy in each block as the dependent variable (Table 2). In the 65% probability of a 9-point reward learning task, no significant differences were observed between groups in the first block (*p* > 0.05). However, significant differences emerged in subsequent blocks (*p* < 0.05), indicating that while the 65% reward probability presented a similar initial difficulty for all participants, the healthy control group demonstrated a faster learning rate.

**Table 2:** Descriptive statistics and ANOVA results for reward probability learning across groups.

**Reward probability**	**Long-term abstinence (standard error)**	**Short-term abstinence (standard error)**	**Healthy control (standard error)**	***F*_(2, 194)_**
**65% probability reward 9 score**				
Block 1	0.596 (0.03)	0.583 (0.02)	0.59 (0.03)	0.071
Block 2	0.635 (0.03)	0.632 (0.02)	0.75 (0.03)	4.427^*^
Block 3	0.702 (0.03)	0.655 (0.03)	0.828 (0.03)	6.609^**^
Block 4	0.755 (0.03)	0.688 (0.03)	0.911 (0.02)	10.897^***^
**75% probability reward 9 score**				
Block 1	0.633 (0.02)	0.595 (0.02)	0.71 (0.03)	4.583^**^
Block 2	0.756 (0.03)	0.689 (0.03)	0.84 (0.04)	4.914^**^
Block 3	0.791 (0.03)	0.718 (0.03)	0.885 (0.03)	6.512^**^
Block 4	0.827 (0.03)	0.747 (0.03)	0.933 (0.02)	9.641^***^
**Combined probabilities**				
Block 1	0.615 (0.019)	0.589 (0.017)	0.65 (0.011)	2.189
Block 2	0.696 (0.023)	0.661 (0.024)	0.795 (0.026)	6.587^**^
Block 3	0.747 (0.028)	0.686 (0.026)	0.857 (0.021)	8.918^***^
Block 4	0.791 (0.026)	0.717 (0.029)	0.922 (0.016)	12.754^***^

^*^p < 0.05, ^**^p < 0.01, ^***^p < 0.001.

In the 75% probability of a 9-point reward learning task, the healthy control group showed significantly higher accuracy than both the long-term and short-term abstinence groups across all four blocks (*p* < 0.05). No significant difference was found between the long-term and short-term abstinence groups (*p* > 0.05), suggesting that the 75% probability stimulus was relatively easy for the healthy control group to learn, resulting in an advantage from the first block. Although the healthy control group exhibited a faster learning rate than the long-term and short-term abstinence groups, this difference was not statistically significant (*p* > 0.05). This further suggests that the 75% probability stimulus was relatively easy for the healthy controls, enabling them to identify the high-probability high-reward character from the first block.

#### 2.2.2 Testing phase

**Figure 4:**
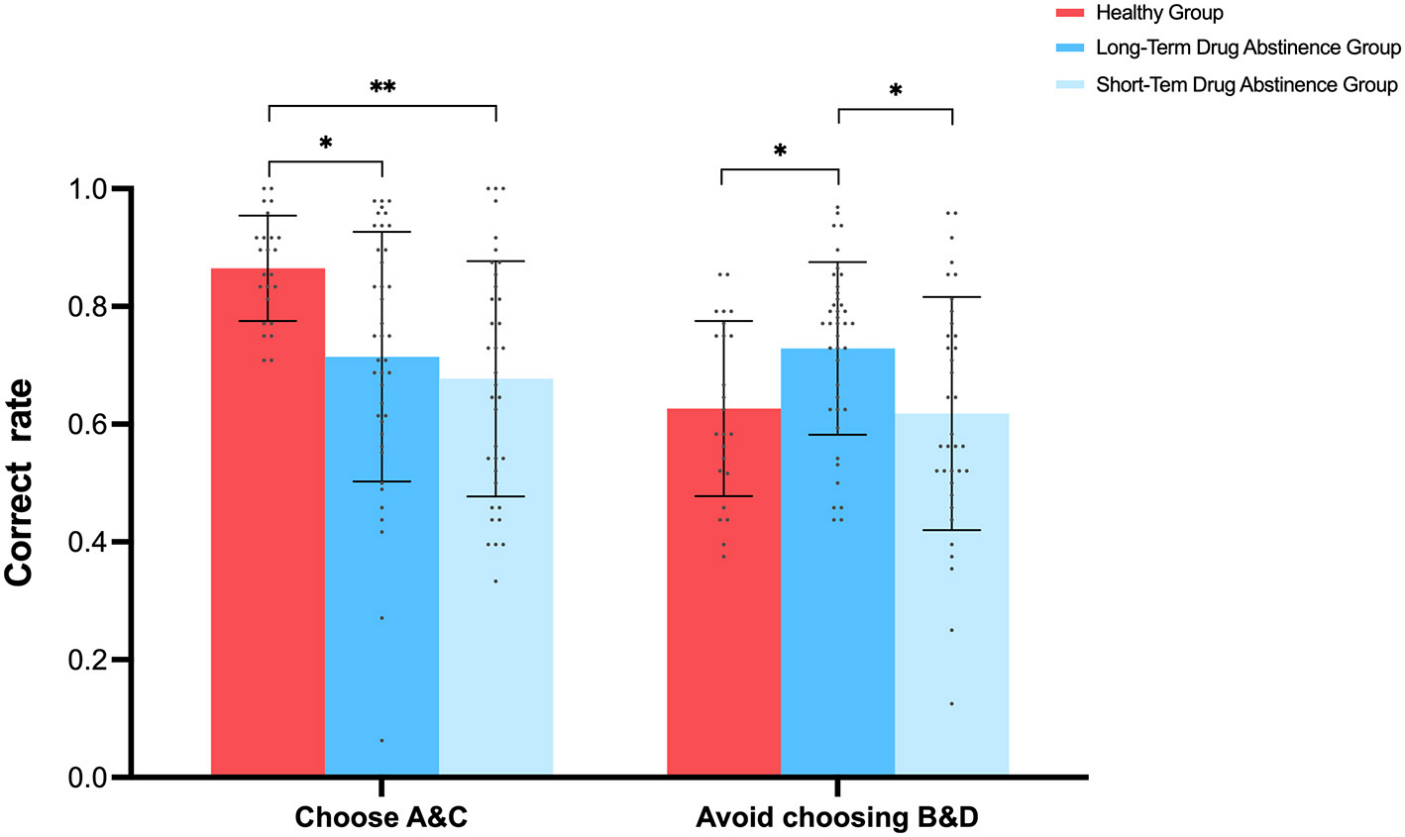
Experimental results from Test 1 showing each group's selection of high-probability high-reward characters (A&C) vs. avoidance of low-probability high-reward characters (B&D) in the reward learning task. *p < 0.05, **p < 0.01.

A repeated-measures ANOVA on accuracy, with two probability discriminations (selecting high-probability high-reward A&C vs. avoiding low-probability high-reward B&D) and three groups (long-term abstinence, short-term abstinence, healthy controls), revealed a significant main effect of probability discrimination, with higher accuracy in selecting A&C compared to avoiding B&D [*F*_(1, 97)_ = 11.832, *p* < 0.001, 95% *CI* = [0.04, 0.14], $\eta_{p}^{2}$ = 0.109]. A significant main effect of group was also observed [*F*_(2, 194)_ = 6.283, *p* < 0.01, $\eta_{p}^{2}$ = 0.115], with *post-hoc* comparisons showing that the healthy control group had significantly higher accuracy than both the long-term and short-term abstinence groups (*p* < 0.05). A significant interaction effect between probability discrimination and group was also found [*F*_(2, 97)_ = 6.724, *p* < 0.01, $\eta_{p}^{2}$ = 0.122]. Simple effects analysis (Figure 4) showed that healthy controls exhibited significantly higher accuracy in selecting A&C (*M* = 0.86, SD = 0.08) compared to avoiding B&D (*M* = 0.62, *SD* = 0.14) [*F*_(1, 97)_ = 18.973, *p* < 0.001, 95% *CI* = [0.13, 0.34]]. Under the A&C condition, the healthy control group showed significantly higher accuracy (*M* = 0.86, *SD* = 0.08) than both the long-term (*M* = 0.71, *SD* = 0.21) and short-term (*M* = 0.67, *SD* = 0.2) abstinence groups [*p* < 0.05, 95% *CI* = [0.034, 0.266], 95% *CI* = [0.069, 0.31]], with no significant difference between the long-term and short-term abstinence groups [*p* > 0.05, 95% *CI* = [−0.141, 0.066]]. Conversely, under the B&D condition, the long-term abstinence group (*M* = 0.729, *SD* = 0.14) performed significantly better than both the healthy control group (*M* = 0.627, *SD* = 0.14) and the short-term abstinence group (*M* = 0.618, *SD* = 0.19) [*p* < 0.05, 95% *CI* = [0.017, 0.2], 95% *CI* = [−0.03, 0.2]], with no significant difference between the healthy control and short-term abstinence groups [*p* > 0.05, 95% *CI* = [−0.09, 0.1]]. Paired samples t-tests revealed no significant difference in accuracy between selecting A&C (M = 0.677, SD = 0.200) and avoiding B&D (M = 0.618, SD = 0.198) in the short-term abstinence group, *t*_(35)_ = 1.177, *p* = 0.247. Similarly, no significant difference was found in the long-term abstinence group between selecting A&C (M = 0.715, SD = 0.212) and avoiding B&D (M = 0.729, SD = 0.147), *t*_(39)_ = −0.321, *p* = 0.750. These results indicate that, unlike the healthy control group, neither short-term nor long-term abstinence groups showed statistically significant differences in accuracy when choosing high-probability high-reward stimuli (A&C) vs. avoiding low-probability high-reward stimuli (B&D). This may suggest a generalized impairment in reward-related decision-making among methamphetamine addicts.

### 2.3 Discussion

This study found that healthy controls exhibited significantly higher accuracy in selecting high-probability high-reward characters (A&C) compared to individuals with methamphetamine addiction, while showing relatively lower accuracy in avoiding low-probability high-reward characters (B&D) during the testing phase. This may be due to healthy controls receiving more frequent positive feedback for selecting high-reward characters, reinforcing their learning and selection of these characters. Conversely, the lower value associated with low-probability high-reward characters (B&D) might lead to reduced cognitive resource allocation, resulting in lower accuracy in this condition (Mather and Sutherland, 2011).

Furthermore, the superior performance of healthy controls in the high-probability high-reward character selection task aligns with previous research showing aberrant striatal responses to natural rewards in individuals with addicted to substances such as methamphetamine (Volkow et al., 2012; Yang et al., 2017; Su and Zheng, 2023). Although no significant differences were observed between addiction groups in discriminating between the two character types, the long-term abstinence group showed slightly higher accuracy than the short-term abstinence group in both the learning and testing phases. The significantly higher accuracy of the long-term abstinence group in the B&D condition during the testing phase suggests a potential degree of recovery in reward processing function with increased abstinence duration, although overall, performance was still inferior to the healthy control group when considering both A&C and B&D conditions, the long-term abstinence group showed significantly higher accuracy than both the healthy control group and the short-term abstinence group under the B&D condition, as shown in Figure 4. However, it is important to note that this superior performance of the long-term abstinence group compared to the healthy control group was specific to the B&D condition (low-probability, high-reward trials), and was not observed in the A&C condition (high-probability, high-reward trials). Additionally, the healthy control group exhibited significantly higher overall accuracy when considering both A&C and B&D conditions [*F*_(2, 194)_ = 6.283, *p* < 0.01, $\eta_{p}^{2}$ = 0.115].

Given the lack of significant differences between the long-term and short-term abstinence groups on key measures, and since abstinence duration was not the primary focus of this study, subsequent experiments will not categorize groups based on abstinence duration. Instead, all addicted individuals with an abstinence duration not exceeding 12 months will be uniformly analyzed as the addiction group. Experiment 1 aimed to verify the existence of aberrant reward processing in individuals with addiction and to investigate the restorative effects of abstinence on reward processing. Considering that addiction is a brain disorder associated with aberrant reward processing, and the close relationship between self-processing and reward processing (Northoff and Bermpohl, 2004), we hypothesize that aberrant reward processing in individuals with addiction may partly stem from aberrant self-processing. Building upon the findings of Experiment 1, Experiment 2 will further investigate whether aberrant self-processing exists in individuals with addiction, potentially providing a new theoretical basis for addiction intervention.

## 3 Experiment 2: aberrant self-processing in individuals with addiction

### 3.1 Methods

#### 3.1.1 Experimental design

A 2 (self-relevance: low, high) × 2 (participant type: addiction, healthy) mixed design was employed, with self-relevance as a within-subject variable and participant type as a between-subject variable. High self-relevance stimuli consisted of the participant's own name and two self-representative characters. Low self-relevance stimuli refer to situations where participants have only minimal interaction with familiar Homo sapiens. We employed the IOS scale developed by Aron et al. (1992) to identify these familiar Homo sapiens names. This scale consists of seven overlapping circles representing varying degrees of overlap between the self and other Homo sapiens. Participants were required to select the circle combination that best reflected their relationship with each individual. We defined “acquaintances” as those with IOS scores below 3 points, indicating extremely low connection or sense of overlap with the participant's self. Only names meeting this criterion were included in the task as low self-relevance stimuli. The IOS scale measurement ensured that stimuli categorized as “acquaintances” were consistently perceived across all participants as having low self-relevance. Participants' gender and name length were strictly matched. To standardize the self-relevance manipulation, all familiar Homo sapiens stimuli were defined as individuals with no substantive social interaction with participants, limited to occasional greetings. Dependent variables included accuracy rates and response times across different judgment types, comprehensively assessing the impact of self-relevance on cognitive processing in both addiction participants and healthy participants.

#### 3.1.2 Participants

This study received ethical approval (Review Number: 671). Experiment 2 used 70 participants: 35 methamphetamine addicts (20 male) from the Changsha City xxx Compulsory Isolation Drug Rehabilitation Center, and 35 healthy controls (20 male) matched for age and education level. All participants were right-handed, had normal or corrected-to-normal vision, no history of alcohol abuse or dependence, no history of brain injury, neurological disease, or psychiatric illness, and had not used any medication that could affect neurological function in the week prior to the experiment. Participants signed informed consent forms before the experiment and received small gifts afterward. The sample size was determined using G^*^Power 3.1.9, with an effect size of *f* = 0.25, α = 0.05, and power (1–β) = 0.95, indicating a minimum of 54 participants were needed. A structured clinical interview (SCID) was used to exclude participants with neurological or psychiatric disorders. Demographic details are in Table 3.

**Table 3:** Demographic variables by group (N = 70).

**Group**	***n***	**Mean age (standard error)**	**Gender ratio (male:female)**	**Education level (standard error)**
Addiction	35	30.54 (0.661)	20:15	10.46 (0.409)
Healthy control	35	31.17 (0.886)	20:15	10.09 (0.417)

No significant differences were found among groups for any demographic variable.

#### 3.1.3 Experimental materials

Self-stimuli consisted of Yi vowel characters from Experiment 1. Before the formal experiment, the experimenter selected four characters from a set of six, guiding participants to assign self-relevant meanings: participants associated two characters (e.g., A and C) with their self-concept and two others (e.g., E and G) with acquaintances. Participants completed this self-linking task within 1 min to ensure personal meaning construction and memory consolidation. The two remaining characters, without assigned meanings, served as neutral controls. To standardize the self-relevance manipulation, all acquaintance stimuli were defined as individuals with whom the participant had no substantive social interaction, limited to occasional greetings.

#### 3.1.4 Experimental procedure

**Figure 5:**
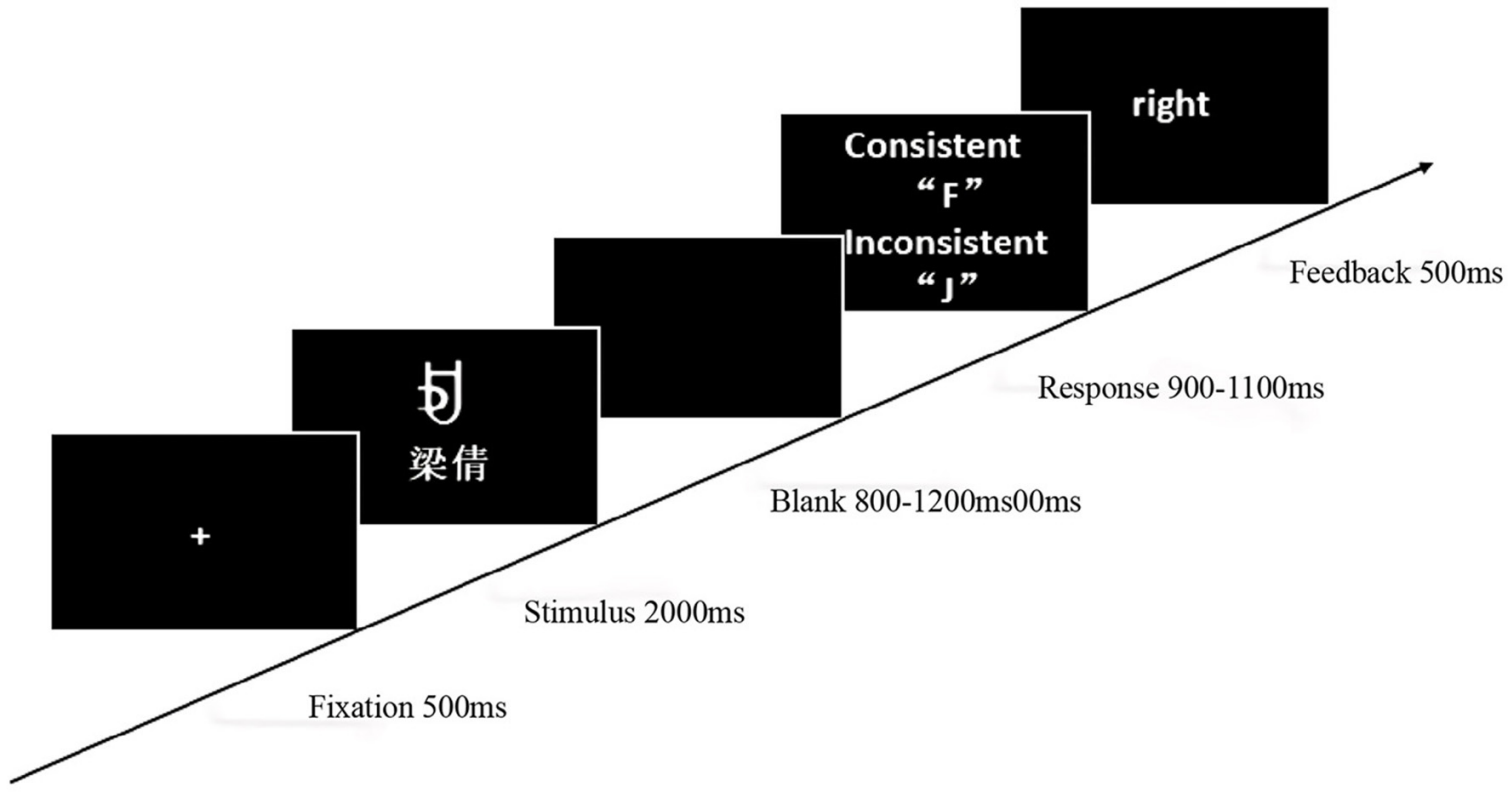
Experiment 2 self-referential judgment task procedure.

The experiment involved learning and testing phases, programmed and recorded using E-Prime 2.0. During the learning phase, participants assigned self-relevant meanings to four characters and memorized them within 1 min. The testing phase comprised three character-name meaning matching conditions: (1) congruent character-name meaning [self (A, C), acquaintance (E, G)]; (2) incongruent character-name meaning [self (B, D), acquaintance (B, D)]; and (3) contradictory character-name meaning [self (E, G), acquaintance (A, C)]. After the fixation point “+” disappeared, a character and name were presented simultaneously for 2,000 ms. After a random blank screen (800–1,200 ms), participants judged the consistency of character meaning and name within 900–1,100 ms and received feedback (Figure 5). The experiment included 360 trials, presented randomly across six blocks, to investigate the modulatory mechanism of self-relevance on cognitive processing.

#### 3.1.5 Data analysis

Judgment types were categorized into two major groups: self-judgment (self-consistent, self-inconsistent, self-contradictory) and acquaintance judgment (acquaintance-consistent, acquaintance-inconsistent, acquaintance-contradictory). One-way ANOVAs were conducted separately for accuracy and reaction time, with judgment type as the independent variable. A 2 (group: addiction, healthy) × 2 (judgment type: self-judgment, acquaintance-judgment) repeated-measures ANOVA was conducted using overall accuracy and reaction time as dependent variables.

### 3.2 Results

**Figure 6:**
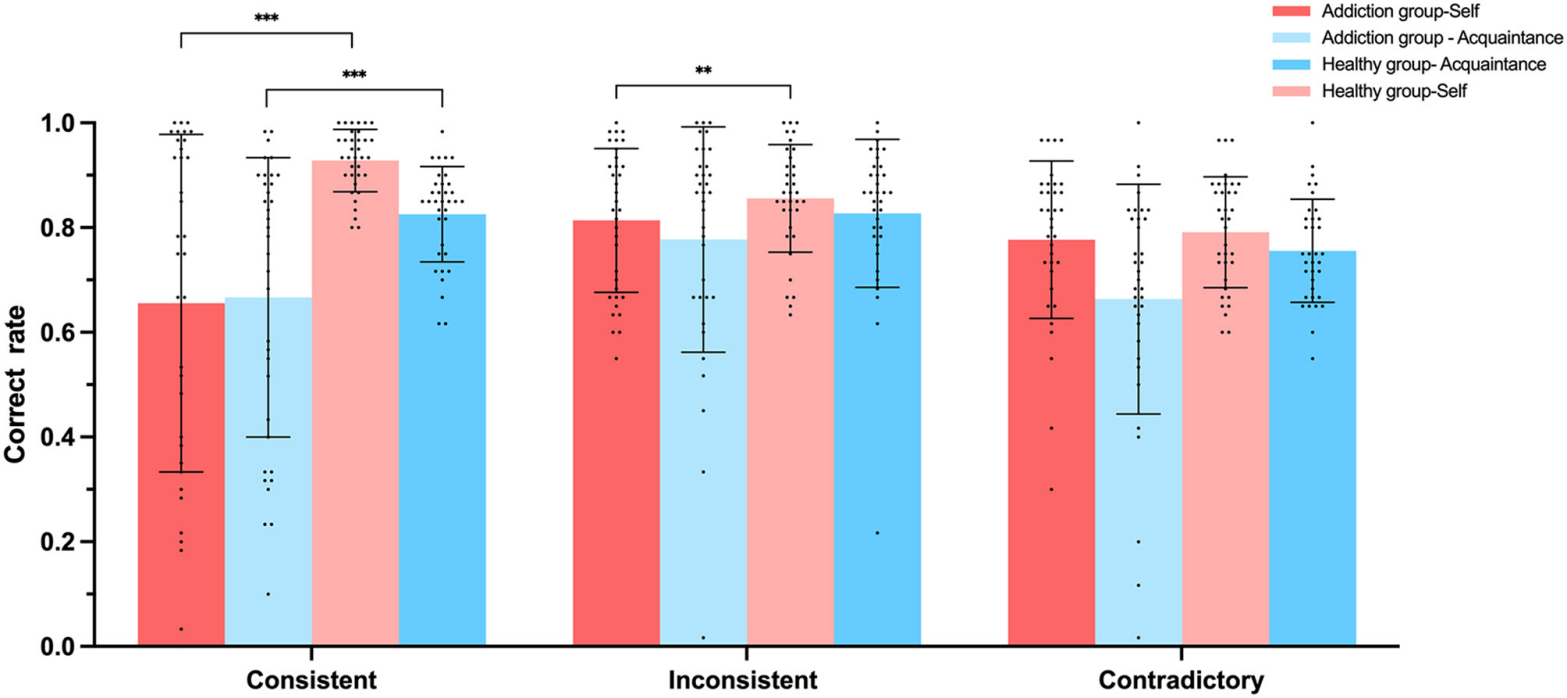
Experiment 2 Accuracy rates (%) of participants under three matching conditions in the self-referential task. **p < 0.01, ***p < 0.001.

**Figure 7:**
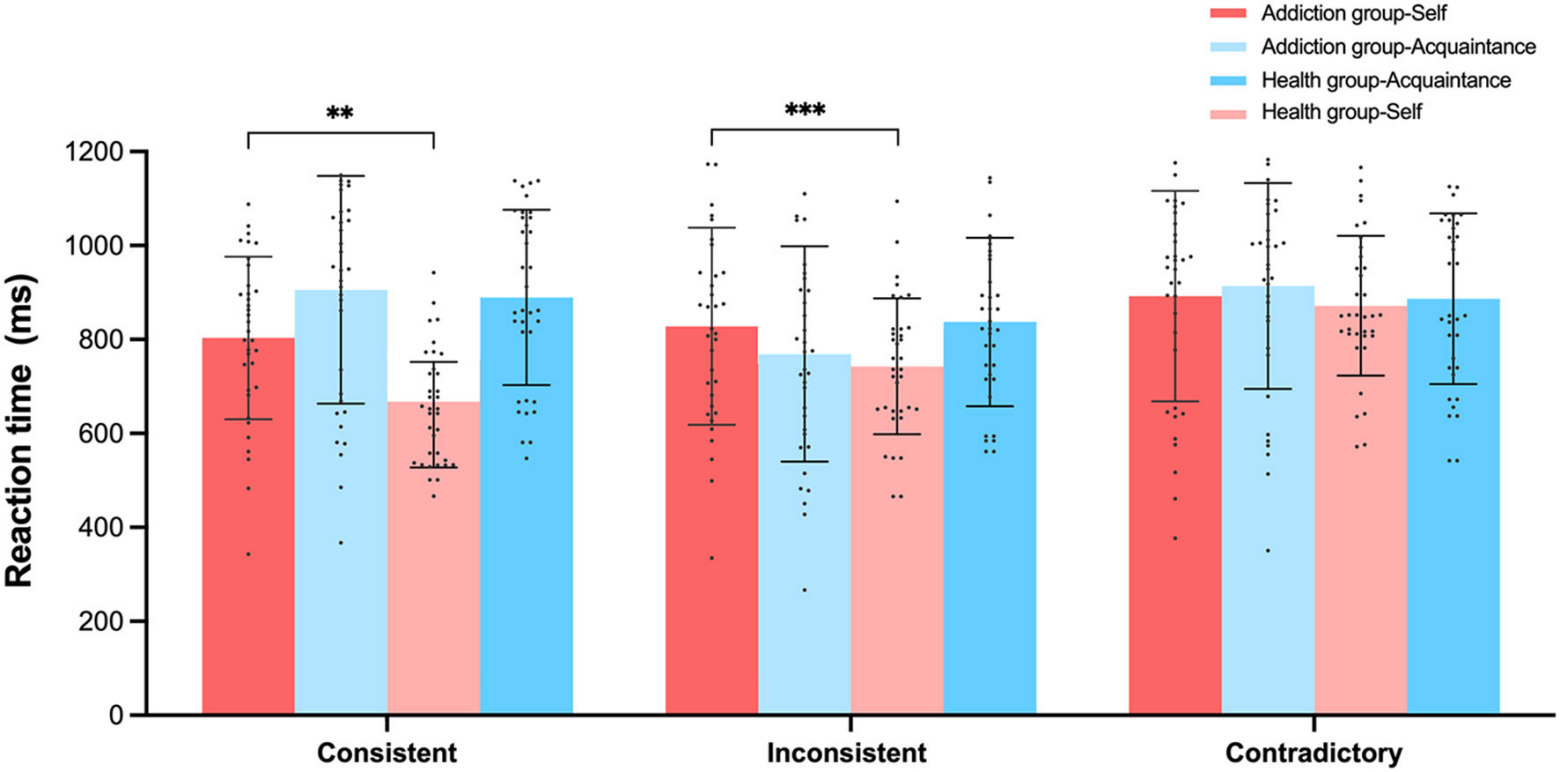
Experiment 2 participants' reaction times (ms) under three matching conditions in the self-referential task. **p < 0.01, ***p < 0.001.

We conducted analysis of variance (ANOVA) with the accuracy rates and reaction times of judgment types as dependent variables, with the results displayed in Figures 6, 7. For self-consistent judgments, the Healthy group-Self (*M* = 0.94, *SD* = 0.04) performed significantly better than the Addiction group-Self (*M* = 0.65, *SD* = 0.32), *F*_(1, 68)_ = 27.215, *p* < 0.001, 95% *CI* = [−0.39, −1.77], $\eta_{p}^{2}$ = 0.286. In terms of self-inconsistent judgment accuracy, the Healthy group-Self (*M* = 0.89, *SD* = 0.07) also outperformed the Addiction group-Self (*M* = 0.81, *SD* = 0.14), *F*_(1, 68)_ = 8.913, *p* < 0.01, 95% *CI* = [−0.13, −0.026], $\eta_{p}^{2}$ = 0.116. Similarly, for acquaintance-consistent judgments, the Healthy group- Acquaintance (*M* = 0.83, *SD* = 0.09) demonstrated significantly higher accuracy than the Addiction group—Acquaintance (*M* = 0.67, *SD* = 0.26), *F*_(1, 68)_ = 11.14, *p* < 0.001, 95% *CI* = [−0.25, −0.006], $\eta_{p}^{2}$ = 0.141. However, no significant differences were found between the Healthy group- Acquaintance (*M* = 0.82, *SD* = 0.14) and the Addiction group—Acquaintance (*M* = 0.77, *SD* = 0.21) for the accuracy of acquaintance-inconsistent judgments, *F*_(1, 68)_ = 11.31, *p* = 0.25, 95% *CI* = [−0.13, −0.037], $\eta_{p}^{2}$ = 0.019.

In terms of reaction times for self-consistent judgments, the Healthy group-Self (*M* = 669.3 ms, *SD* = 71.9) responded significantly faster than the Addiction group-Self (*M* = 803.3 ms, *SD* = 173.1), *F*_(1, 68)_ = 17.897, *p* < 0.001, 95% *CI* = [70.79, 197.21], $\eta_{p}^{2}$ = 0.208. For self-inconsistent judgment reaction times, the Healthy group-Self (*M* = 744.7 ms, *SD* = 102.1) again responded significantly faster than the Addiction group-Self (*M* = 828.03 ms, *SD* = 209.7), *F*_(1, 68)_ = 4.459, *p* < 0.05, 95% *CI* = [4.57, 161.9], $\eta_{p}^{2}$ = 0.062.

As shown in Table 4, we also conducted a repeated measures ANOVA on the accuracy rates across two groups (addiction and healthy) and two judgment types (self-judgment and acquaintance judgment). The results revealed a significant main effect for judgment type, with self-judgment (*M* = 0.81, *SD* = 0.133) demonstrating significantly higher accuracy than acquaintance judgment (*M* = 0.75, *SD* = 0.07), *F*_(1, 68)_ = 28.976, *p* < 0.001, 95% *CI* = [0.038, 0.082], $\eta_{p}^{2}$ = 0.299. A significant main effect between groups was also observed, with the healthy group (*M* = 0.83, *SD* = 0.07) showing higher accuracy than the addiction group (*M* = 0.72, *SD* = 0.16), *F*_(1, 68)_ = 17.956, *p* < 0.001, 95% *CI* = [0.06, 0.16], $\eta_{p}^{2}$ = 0.299. However, the interaction between group and judgment type was not significant.

**Table 4:** Descriptive statistics of accuracy rates and reaction times for the addiction group and healthy control group in judgment tasks.

**Group**	**Judgment type**	**Accuracy (standard deviation)**	**Reaction time (standard deviation)**
Addiction	Self	0.749 (0.157)	841.203 (190.563)
	Acquaintances	0.702 (0.161)	888.639 (217.924)
Healthy control	Self	0.876 (0.054)	761.939 (73.388)
	Acquaintances	0.803 (0.074)	872.639 (158.502)

A repeated measures analysis of variance (ANOVA) was conducted on the reaction times for all judgments across two groups (addiction and healthy) and two judgment types (self-judgment and acquaintance judgment). The analysis revealed a significant main effect for judgment type, with self-judgment reaction times being significantly faster than acquaintance judgment reaction times, *F*_(1, 68)_ = 34.293, *p* < 0.001, 95% *CI* = [43.3, 88.1], $\eta_{p}^{2}$ = 0.335. There was also a significant interaction between group and judgment type, *F*_(1, 68)_ = 16.046, *p* < 0.01, $\eta_{p}^{2}$ = 0.191.

Further simple effects analysis showed that within the different judgment types, the healthy control group exhibited significantly faster reaction times for self-judgment compared to acquaintance judgment, *F*_(1, 68)_ = 5.273, *p* < 0.05, 95% *CI* = [−142.3, −79.02], $\eta_{p}^{2}$ = 0.072. In contrast, the addiction group did not show a significant difference between self-judgment and acquaintance judgment reaction times, *F*_(1, 68)_ = 1.712, *p* = 0.19, 95% *CI* = [−52.4, 10.9], $\eta_{p}^{2}$ = 0.025.

Moreover, across the different participant groups, the healthy control group had significantly faster reaction times for self-judgment compared to the addiction group, *F*_(1, 68)_ = 48.6, *p* < 0.001, 95% *CI* = [−148.14, −10.38], $\eta_{p}^{2}$ = 0.417. However, there were no significant differences in reaction times for acquaintance judgments between the two groups, *F*_(1, 68)_ = 0.62, *p* > 0.05, 95% *CI* = [−74.5, 95.8], $\eta_{p}^{2}$ = 0.001.

### 3.3 Discussion

The significant main effect of judgment type on accuracy indicates that both healthy controls and individuals with addiction exhibited higher accuracy when stimuli were highly self-relevant. This supports the notion that individuals with addiction experience self-referential effects (Yin et al., 2019). Self-referential processing refers to the tendency of individuals to process and remember self-related information more efficiently and deeply than information related to others (Newlin and Renton, 2010). This effect is attributed to the unique activation of brain regions associated with self-processing, such as the medial prefrontal cortex (MPFC), which plays a crucial role in self-referential cognition (D'Argembeau, 2013).

In our study, both healthy individuals and those with addiction demonstrated self-referential effects. However, the magnitude of this effect was reduced in the addiction group, suggesting potential impairments in self-processing among individuals with addiction. This conclusion is supported by the finding that individuals with addiction exhibited significantly lower accuracy (M = 0.72, SD = 0.16) compared to the healthy control group (M = 0.83, SD = 0.07), *F*_(1, 68)_ = 17.956, *p* < 0.001, 95% CI = [0.06, 0.16], $\eta_{p}^{2}$ = 0.299. The absence of a significant interaction effect further implies that individuals with addiction demonstrate generally lower accuracy in processing self- or acquaintance-linked characters, indicating potential deficits in self-processing (Denny et al., 2012).

Regarding reaction time, the significant interaction between group and judgment type, along with subsequent simple effects analysis, reveals that healthy controls showed significantly faster reaction times for self-relevant judgments compared to acquaintance-relevant judgments. This difference was not observed in the addiction group. Furthermore, healthy controls demonstrated significantly faster reaction times for self-relevant judgments compared to the addiction group, while no significant group differences were found for acquaintance-relevant judgments.

Overall, these findings confirm the existence of self-referential effects in individuals with addiction but also highlight relatively weaker self-processing abilities (Pankow et al., 2016). Building on the results of Experiments 1 and 2, Experiment 3 will further investigate the relationship between reward processing and self-processing in individuals with addiction. Specifically, it will explore how deficits in self-processing may modulate reward sensitivity in addiction, providing deeper insights into the interplay between self and reward processing. This line of inquiry has the potential to offer novel perspectives for addiction intervention.

## 4 Experiment 3: aberrant self-processing in individuals with addiction

### 4.1 Methods

#### 4.1.1 Experimental design

Experiment 3 employed a 2 (participant type: addiction, healthy) × 4 (self-linked reward: high self-relevance—high probability reward, high self-relevance—low probability reward, low self-relevance -high probability reward, low self-relevance—low probability reward) mixed design to investigate the interactive effects of self-linking and reward level on decision-making behavior in individuals with addiction and healthy controls. Participant type was a between-subject variable, while self-linked reward was a within-subject variable. The dependent variable was the accuracy of selecting high-probability high-reward options. By examining decision-making performance under different self-linking levels and reward conditions, the study aimed to analyze the cognitive processing characteristics of individuals with addiction in depth. The manipulation of self-relevance was consistent with Experiment 2 to ensure experimental paradigm consistency.

#### 4.1.2 Participants

In Experiment 3, a sample size of 70 participants was newly recruited, comprising 35 methamphetamine addicts from the Changsha City xxx Compulsory Isolation Drug Rehabilitation Center (20 male) and 35 matched healthy controls (20 male). It should be noted that the participants in Experiment 3 were distinct from those in Experiment 2, ensuring the independence of the two experiments. This study received ethical approval (Review Number: 671). The sample size was determined using G^*^Power 3.1.9 (Heinrich-Heine-Universität Düsseldorf, Germany) analysis, with an effect size of *f* = 0.25, α = 0.05, and power (1–β) = 0.95, indicating a minimum of 36 participants were needed. All addiction participants met DSM-5 criteria and were screened for neurological or psychiatric disorders using the Structured Clinical Interview for DSM-5 (SCID). Participants were right-handed, had normal or corrected-to-normal vision, no history of alcohol abuse or dependence, brain injury, neurological disease, or psychiatric illness, and had not used neurologically active medication in the week prior to the experiment. Participants provided informed consent after a thorough explanation of the study procedures. Due to institutional regulations, cash compensation was not possible; participants received prizes based on their final scores. Demographic characteristics are detailed in Table 5.

**Table 5:** Demographic variables by group (*N* = 70).

**Group**	***n***	**Mean age (standard error)**	**Gender ratio (male:female)**	**Education level (standard error)**
Addiction	35	29.63 (0.778)	20:15	10 (0.482)
Healthy control	35	31.66 (0.883)	20:15	9.94 (0.445)

No significant differences were found among groups for any demographic variable.

#### 4.1.3 Experimental materials

**Figure 8:**
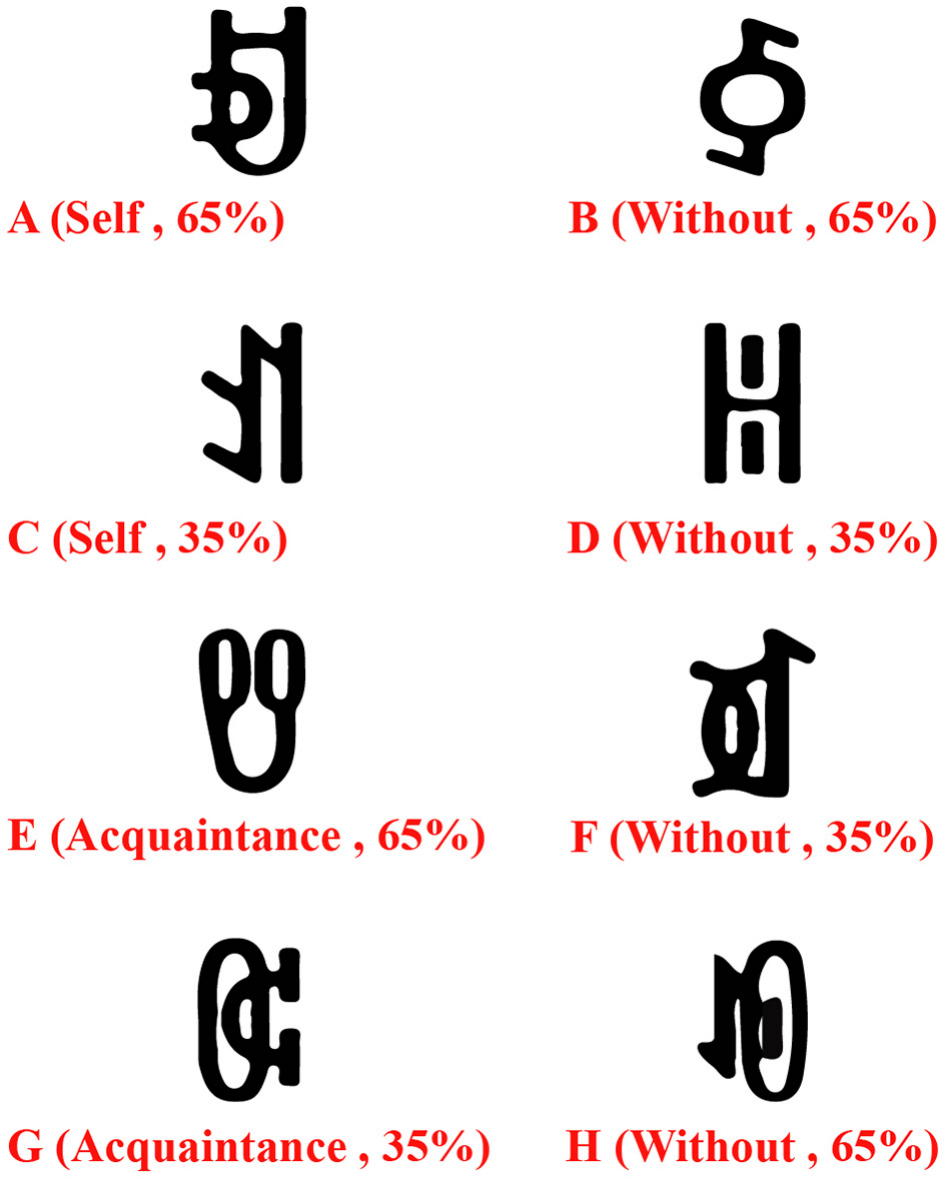
The stimulus materials for Experiment 3 involved linking 4 out of the 8 Yi vowel characters with either “self” or “acquaintance”.

Experiment 3 reused the eight Yi vowel characters from Experiment 1. Participants first assigned self-relevance to four characters (e.g., A and C as self, E and G as acquaintances) within 1 min. Based on Experiment 1, where 75% reward probability proved too easy for healthy controls but 65% offered a suitable challenge for both groups (with faster learning in healthy controls), a 65% reward probability was used to control difficulty and assess how self-processing modulates reward processing, four conditions (Figure 8) were created: one self-relevant character had a 65% chance of a 9-point reward, the other a 35%; the same applied to acquaintance-relevant characters. The remaining four unassigned characters were randomly paired with the assigned characters, ensuring each pair maintained a constant total probability of 1 for a 9-point reward.

#### 4.1.4 Experimental procedure

**Figure 9:**
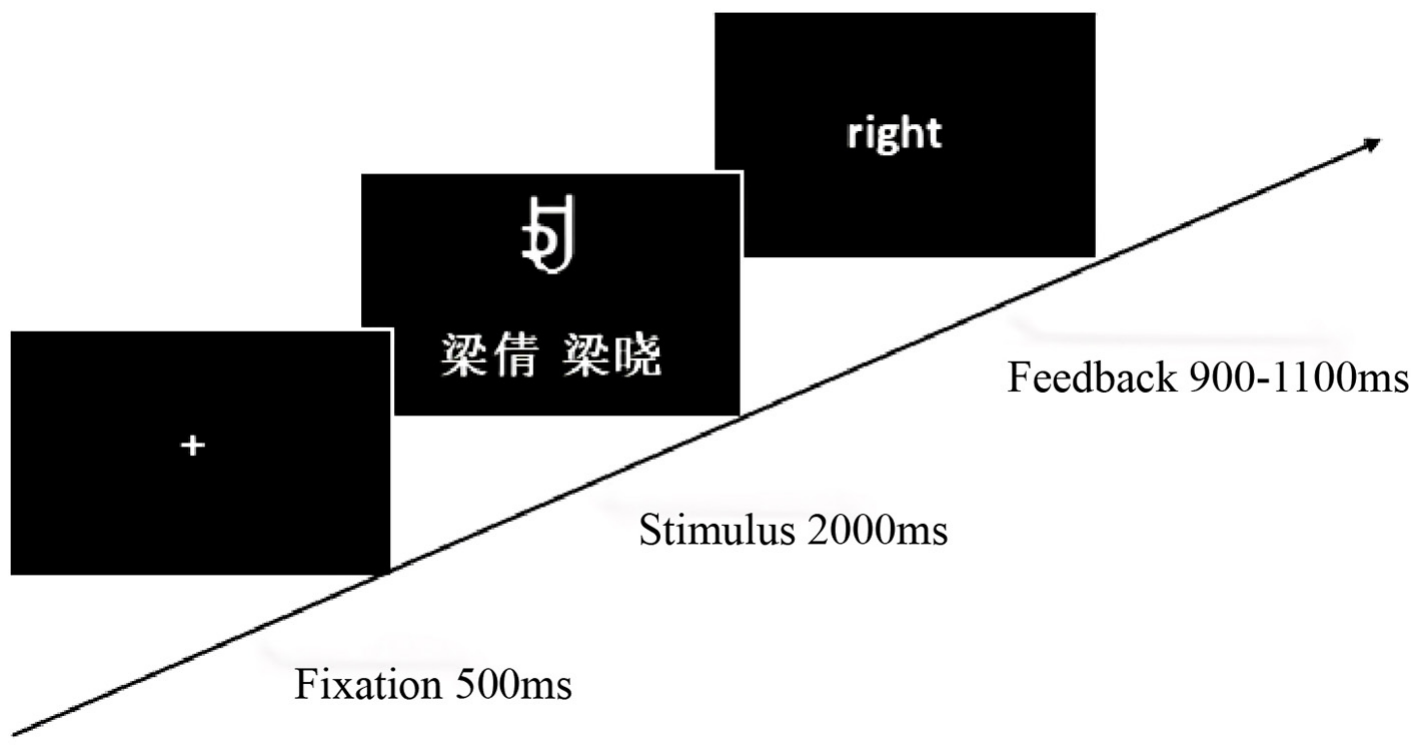
Experiment 3 self-association link test procedure.

This experiment utilized E-Prime 2.0 for programming and data recording, consisting of a learning phase and a formal experiment. In the learning phase, four of the eight Yi vowel characters were randomly presented, and participants assigned self-relevance within 1 min. The other four characters were not shown before the formal experiment. A subsequent association test (Figure 8), using only character-name matching (to prevent interference from the other four characters), required participants to achieve 100% accuracy to proceed to the reward learning task (Figure 9). This ensured that participants had firmly linked the characters to their self or acquaintance concepts before proceeding. Additionally, we imposed a time limit on the association task to prevent overthinking and strategic selection, ensuring the spontaneity and genuineness of the assigned meanings. We also averaged the presentation of the characters across all blocks to avoid any potential bias. The formal experiment then began, with a reward learning task identical to Experiment 1. A reward processing test followed, differing from Experiment 1 by randomly presenting character pairs: A(B, F), C(D, H), E(B, F), G(D, H). Participants needed to select characters with a 65% probability of receiving a high reward while avoiding characters with a 35% probability of receiving a low reward to maximize their total reward points.

#### 4.1.5 Data analysis

The primary dependent variables were the performance of both groups (addiction group and healthy control group) across the stages of the reward learning experiment and the accuracy rates for characters associated with self-relevant information. First, an independent samples *t*-test was conducted to compare the overall performance of the two groups during the learning phase. Second, to test the effect of different reward types on learning, a 2 (between-subjects factor: addiction group vs. healthy control group) × 4 (within-subjects factor: self-65%, acquaintance-65%, self-35%, acquaintance-35%) repeated measures analysis of variance (ANOVA) was performed on the accuracy rate data for characters associated with self-relevant information. *Post-hoc* comparisons were conducted using Bonferroni-corrected multiple comparisons. All statistical analyses were performed using SPSS software, with a significance level set at α = 0.05.

### 4.2 Results

In the reward learning experiment, a one-way ANOVA (see Table 6) was conducted on the accuracy rates of both groups across all stages and for characters associated with self-relevance. Results indicated a significant difference between groups across all four blocks in the 65% (9-point reward) learning task, with the healthy control group exhibiting significantly higher accuracy than the addiction group (*ps* < 0.01).

**Table 6:** Descriptive statistics and ANOVA results for reward task performance.

**Probability of self-reward**	**Addiction group (standard error)**	**Healthy control group (standard error)**	***F*_(1, 68)_**
Block 1	0.579 (0.02)	0.687 (0.02)	17.963^***^
Block 2	0.694 (0.02)	0.783 (0.02)	10.018^**^
Block 3	0.742 (0.02)	0.85 (0.02)	13.686^***^
Block 4	0.76 (0.03)	0.936 (0.01)	29.045^***^
Self-65%	0.578 (0.04)	0.883 (0.02)	62.92^***^
Acquaintance-65%	0.675 (0.03)	0.836 (0.03)	15.256^***^
Self-35%	0.593 (0.04)	0.737 (0.02)	11.239 ^***^
Acquaintance-35%	0.828 (0.03)	0.672 (0.03)	14.785^***^

^**^p < 0.01, ^***^p < 0.001.

A 2 (between-subjects: addiction group vs. healthy control group) × 4 (within-subjects: self-65%, acquaintance-65%, self-35%, acquaintance-35%) repeated measures ANOVA was performed on the accuracy rates for characters associated with self-relevant information. This analysis revealed a significant main effect of self-relevant reward, *F*_(3, 204)_ = 8.165, *p* < 0.001, $\eta_{p}^{2}$ = 0.107. *Post-hoc* comparisons (Bonferroni corrected) showed that accuracy for self-65% (*M* = 0.77, *SD* = 0.08) was significantly higher than for acquaintance-65% (*M* = 0.70, *SD* = 0.08), 95% *CI* = [0.024, 0.12], and self-35% (*M* = 0.66, *SD* = 0.08), 95% *CI* = [0.063, 0.16] (*ps* < 0.05). However, there was no significant difference between self-65% and acquaintance-35% (M = 0.75, SD = 0.08), *ps* = 0.26, 95% *CI* = [−0.022, 0.081]. Accuracy for acquaintance-35% was significantly higher than for self-35%, 95% *CI* = [0.032, 0.13], but not significantly different from acquaintance-65% (*ps* > 0.05, 95% *CI* = [−0.06, 0.09]).

There was also a significant main effect of group, with the healthy control group (*M* = 0.78, *SD* = 0.08) showing significantly higher accuracy than the addiction group (*M* = 0.66, *SD* = 0.08), *F*_(1, 68)_ = 17.185, *p* < 0.001, 95% *CI* = [0.059, 0.168], $\eta_{p}^{2}$ = 0.202. A significant interaction effect was observed between self-relevant reward and group, *F*_(3, 204)_ = 28.081, *p* < 0.001, $\eta_{p}^{2}$ = 0.3. Simple effects analysis revealed that the healthy control group showed significantly higher accuracy than the addiction group across the other three levels of self-relevant reward (*ps* < 0.01). However, in the condition of acquaintance-35% (35% chance of a 9-point reward), the healthy control group (*M* = 0.67, *SD* = 0.17) showed significantly lower accuracy than the addiction group (*M* = 0.82, *SD* = 0.16), *p* < 0.001, 95% *CI* = [−0.238, −0.075]. Furthermore, the healthy control group's accuracy was significantly higher under the self-65% condition (*M* = 0.88, *SD* = 0.089) compared to self-35% (*M* = 0.73, *SD* = 0.14), *p* < 0.001, 95% *CI* = [0.08, 0.22], and acquaintance-35% (*M* = 0.67, *SD* = 0.17), p < 0.001, 95% *CI* = [0.14, 0.27], but not significantly different from acquaintance-65% (*M* = 0.83, *SD* = 0.15), *p* > 0.05, 95% *CI* = [−0.10, 0.10]. Accuracy under the acquaintance-65% condition was significantly higher than under the acquaintance-35% condition, *p* < 0.001, 95% *CI* = [0.11, 0.21].

The addiction group showed significantly higher accuracy under the acquaintance-35% condition (*M* = 0.82, *SD* = 0.16) compared to self-65% (*M* = 0.67, *SD* = 0.18), *p* < 0.001, 95% *CI* = [0.07, 0.23]; self-35% (M = 0.59, SD = 0.21), p < 0.001, 95% CI = [0.15, 0.31]; and acquaintance-65% (*M* = 0.57, *SD* = 0.20), *p* < 0.001, 95% *CI* = [0.16, 0.33]. Additionally, the addiction group's accuracy under the self-65% condition was significantly higher than under the acquaintance-65% condition, *p* < 0.001, 95% *CI* = [0.02, 0.17], while the difference between self-35% and acquaintance-65% was not significant [*p* > 0.05, 95% *CI* = [−0.04, 0.06]].

### 4.3 Discussion

**Figure 10:**
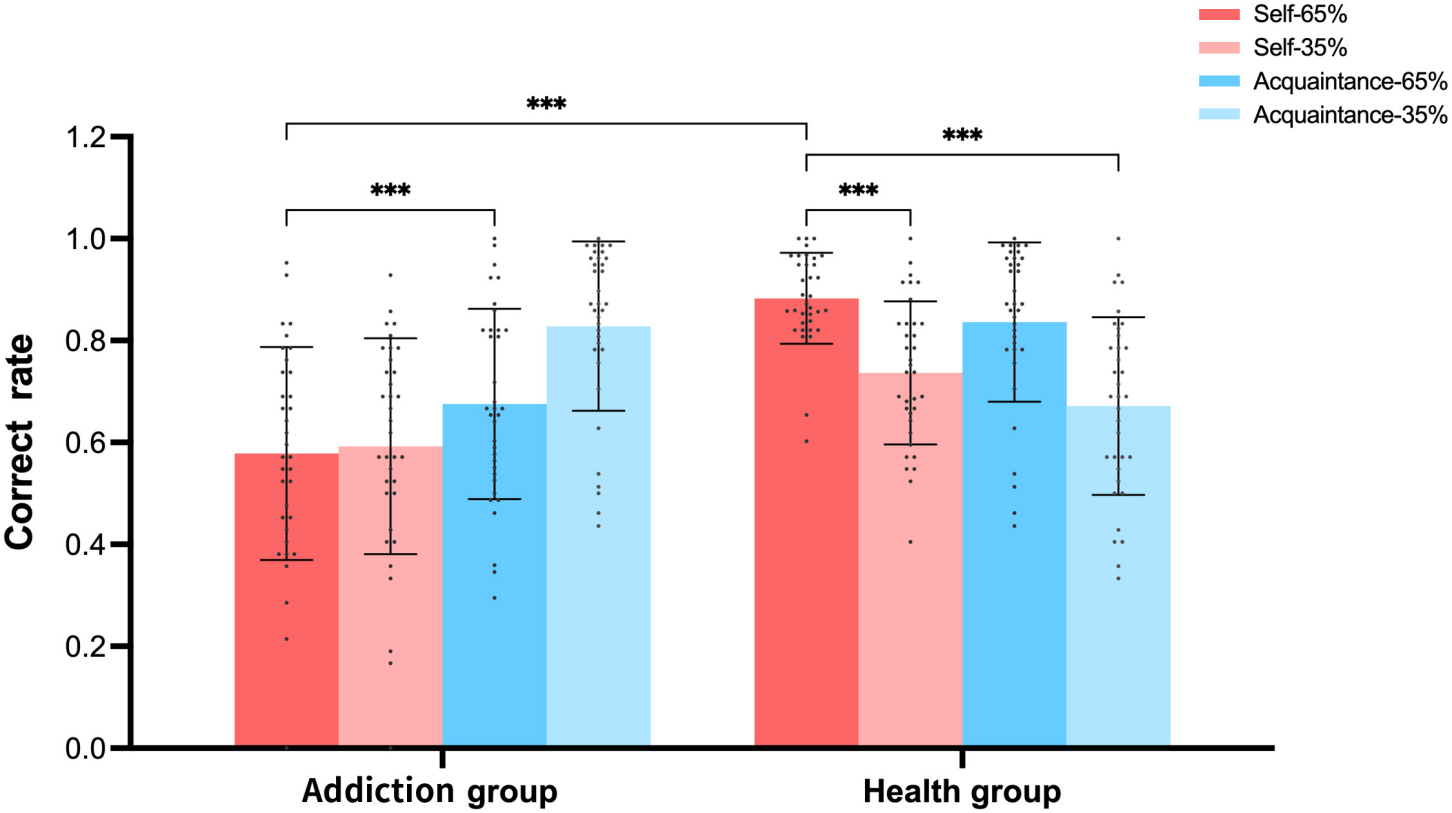
Accuracy rates of each group in the self-associated reward characters task in Experiment 3. ***p < 0.001.

As shown in Figure 10, under high reward probability conditions, the accuracy for self-referential characters among healthy participants (0.89) was slightly higher than that for acquaintance-referential characters (0.84), although this difference did not reach statistical significance. This finding is consistent with previous research (Sui et al., 2015), supporting the notion that self-referencing enhances reward processing in healthy individuals. Similarly, among individuals with addiction, self-referential accuracy was observed to be higher than that of acquaintance-referential characters, indicating that even in individuals with addiction, self-referential processing still exerts a certain degree of enhancement effect on cognitive performance, which is consistent with the self-referential effects observed in healthy individuals. However, the magnitude of this effect is reduced in the addiction group. This is supported by literature indicating that self-referential processing can enhance memory and cognitive performance by integrating new information with existing self-concept representations (Garoff et al., 2005; Serbun et al., 2011). For example, research has shown that self-referential processing is associated with increased activation in brain regions related to self-processing, such as the medial prefrontal cortex (MPFC), which plays a crucial role in self-referential cognition (Qin and Northoff, 2011; D'Argembeau, 2013; Moeller and Goldstein, 2014; Ceceli et al., 2022). Individuals with addiction may still utilize self-referential processing to some extent, but the efficiency and effectiveness of this processing may be impaired due to neurobiological changes associated with addiction (Antons et al., 2023; Yan et al., 2023).

Notably, individuals with addiction displayed a higher accuracy when processing characters with low reward and low self-relevance, this could be interpreted as a form of attentional bias or heightened sensitivity to certain types of stimuli, which may be linked to the neurobiological changes associated with addiction. Research has shown that individuals with addiction often exhibit attentional biases toward drug-related stimuli, which can influence their cognitive processing and behavior (Field and Cox, 2008; Nie et al., 2016). This heightened sensitivity to specific stimuli may reflect an adaptive mechanism in response to the altered reward processing and neurobiological profiles in individuals with addiction (Moretta and Buodo, 2021). Previous studies have indicated that individuals with addiction exhibit lower activation levels in self-referential processing-related brain regions than healthy individuals (De Greck et al., 2010), which may explain the unusual performance of addicted participants in the current study. We hypothesize that this high accuracy under low reward and low self-relevance conditions may result from abnormalities in self-processing and reward processing in the addicted population.

In summary, the results of this study demonstrate a general enhancement effect of self-processing on reward processing; however, this enhancement effect is relatively weaker in populations with addiction. This finding supports Northoff and Hayes's (2011) proposed parallel model of the interaction between self-processing and reward processing, providing new empirical evidence for understanding the cognitive mechanisms of addiction.

## 5 General discussion

This study sought to elucidate the interplay between reward processing and self-referential processing in individuals with methamphetamine addiction undergoing abstinence. Across three experiments, we investigated whether addiction-related deficits in reward processing extend to the processing of self-relevant information and how these processes interact. Experiment 1 revealed impaired reward learning in individuals with methamphetamine addiction, with limited improvement observed with short-term abstinence, highlighting the persistent nature of these deficits. Experiment 2 demonstrated a blunted sensitivity to self-relevant stimuli in the addiction group compared to healthy controls, suggesting a disruption in the salience of self-related cues. Critically, Experiment 3 showed that while self-relevance modulated reward learning in both the methamphetamine addiction group and the healthy control group, the magnitude of this effect was significantly attenuated in individuals with addiction, indicating a compromised integration of self and reward processes.

### 5.1 Impairment of reward processing in individuals with addiction

Experiment 1 revealed impaired reward processing in individuals with addiction compared to healthy controls. This impairment was significant across all conditions and particularly evident at reward probabilities of 65% and 75%. Healthy controls exhibited significantly higher accuracy rates in high-reward probability tasks than both long-term and short-term abstinent groups. Although the long-term abstinent group showed slightly better performance than the short-term abstinent group, both remained significantly below the performance levels of the healthy control group. These findings suggest limited restorative effects of abstinence on reward processing in individuals with addiction.

Consistent with prior research (Yang et al., 2015, 2017), we observed impaired reward processing in individuals with addiction, likely stemming from structural and functional alterations within key reward-related brain regions, including the basal ganglia, extended amygdala, and prefrontal cortex (Volkow et al., 2012; Koob and Le Moal, 2008; Bechara and Damasio, 2005). For example, the nucleus accumbens plays a crucial role in the experience of reward and motivation (Schultz, 2016), while the dorsal striatum is involved in forming habits and routine behaviors (Volkow et al., 2012). The extended amygdala regulates stress responses and negative emotions, which can drive drug-seeking behavior during withdrawal (Koob and Le Moal, 2008). The prefrontal cortex is responsible for executive functions, and its dysfunction can lead to impaired decision-making and increased impulsivity in individuals with addiction (Bechara and Damasio, 2005). These regions are critical for assigning value to stimuli and guiding goal-directed behavior. This impairment is a key finding, underscoring the pervasive impact of addiction on fundamental reward circuitry. The reduced activity in these regions may lead to a decreased ability to experience pleasure from natural rewards, contributing to the cycle of addiction (Volkow et al., 2012).

### 5.2 Impaired sensitivity to self-relevant information processing in individuals with addiction

Experiment 1 demonstrated altered reward processing in individuals with addiction. However, whether a self-referential effect was present in this population remained an open question. Given the established relationship between reward and self-processing, Experiment 2 investigated whether self-processing, a process often intertwined with reward processing, was similarly affected in individuals with addiction. Demonstrating a self-referential effect in this population would significantly enhance our understanding of the interplay between self and reward processing in the context of addiction. Furthermore, such findings could inform the development of targeted interventions aimed at improving addiction-related behaviors. To maintain homogeneity and focus on the presence of a self-referential effect, Experiment 2 included only participants with short-term abstinence.

Extending the findings from Experiment 1, our results suggest that addiction also impacts the processing of self-relevant information. Specifically, compared to healthy controls, the addiction group exhibited significantly lower accuracy across all four conditions (self-consistent, self-inconsistent, acquaintance-consistent, and acquaintance-inconsistent), indicating a general reduction in their ability to process self-relevant information accurately. This finding contrasts with previous research demonstrating that individuals typically exhibit stronger self-referential effects for highly self-relevant information (Wilson et al., 2013; Sel et al., 2019; Yin et al., 2021). The reduced accuracy in the addiction group may be related to previous findings of reduced activation in self-related brain regions (Denny et al., 2012; Frings and Wentura, 2014), potentially weakening the association between highly self-relevant stimuli and the core self, reflecting altered self-processing.

The observed blunted sensitivity to self-relevant stimuli in Experiment 2 may reflect reduced activation or altered functional connectivity within brain regions associated with self-referential processing, such as the medial prefrontal cortex and posterior cingulate cortex (Denny et al., 2012; Frings and Wentura, 2014). This neural disruption could indicate a weakened association between self-related stimuli and the individual's core sense of self, potentially contributing to the mechanisms by which addiction undermines self-identity and motivation.

### 5.3 Weakened enhancement of reward processing by self-relevant information in individuals with addiction

Experiment 2 indicated impaired self-referential processing in individuals with addiction. Typically, self-processing enhances various cognitive functions such as attention, memory, and decision-making (Yin et al., 2019; Zhu and Zhan, 2019). However, the modulatory effect of self-processing on reward processing in individuals with addiction remained unclear. Therefore, Experiment 3 was designed to investigate whether self-processing enhances or diminishes reward processing in individuals with addiction and compare this effect to healthy controls. This comparison would help us further elucidate the relationship between self and reward and inform strategies for correcting addictive behaviors.

The results of Experiment 3 indicated that priming self-processing enhanced reward processing in healthy controls: their learning efficiency was higher when high-reward, high-probability characters were self-associated compared to when they were associated with an acquaintance. In contrast, while the addiction group also showed enhanced reward processing with self-association (self-65% accuracy was significantly higher than acquaintance-65% accuracy, and acquaintance-65% accuracy was not significantly different from self-35% accuracy), this enhancement was less pronounced than in healthy controls.

The attenuated modulation of reward learning by self-relevance in the addiction group provides further evidence for a disruption in the interaction between these two processes. While self-relevant cues still influenced reward learning in individuals with addiction, the effect was significantly weaker than in healthy controls. This suggests that the capacity for self-related information to modulate reward-based decisions may be diminished in individuals with methamphetamine addiction, potentially offering a target for interventions aimed at improving decision-making related to reward.

Interestingly, the addiction group showed significantly higher accuracy for acquaintance-associated characters with a 35% probability of receiving a 9-point reward than the healthy control group. This may suggest that while self-processing is weakened in the addiction group, their processing of low self-relevant information is more thorough. In contrast, the healthy group's preference for high self-relevant and rewarding information may diminish the impact of low-reward processing on their self-processing. Given that both reward and self-processing are superior in the healthy control group, they may be more likely to focus on stimuli with both high reward and high self-relevance.

Prior research highlights the substantial role of attentional bias in reward and self-processing (Poh et al., 2019; Sel et al., 2019; Zhao et al., 2018). This attentional bias, which favors information leading to greater rewards, may explain the healthy control group's tendency to focus on high-reward, high self-relevant stimuli while neglecting low-reward, low self-relevant stimuli. This selective attention aligns with Mather and Sutherland (2011) attentional arousal theory, positing that individuals allocate more cognitive resources to high-value information. Consequently, the observed results may reflect a strategic focus by the healthy group on high-reward, high self-relevant characters, leading to diminished memory for low-reward, low self-relevant characters (Wu et al., 2014).

This study has several limitations. First, the sample size was relatively small, which may have limited the statistical power to detect subtle effects. Second, the study focused on methamphetamine addiction, and the findings may not generalize to other forms of addiction. Third, the study did not investigate the neural mechanisms underlying the observed behavioral effects. Future research should address these limitations by using larger sample sizes, examining other forms of addiction, and incorporating neuroimaging techniques to investigate the neural correlates of self-processing and reward processing in individuals with addiction. Additionally, future studies should explore the potential for interventions targeting self-processing to improve reward processing and reduce addictive behaviors.

## 6 Conclusion

This study's findings demonstrate that: (1) individuals with methamphetamine addiction exhibit impaired reward processing compared to healthy controls, with abstinence showing limited restorative effects; (2) individuals with methamphetamine addiction demonstrate abnormalities in self-processing; and (3) priming self-processing modulates reward processing in individuals with methamphetamine addiction, although this effect is attenuated compared to healthy controls. These findings support a parallel model of interaction between self-processing and reward processing.

## Data Availability

The raw data supporting the conclusions of this article will be made available by the authors, without undue reservation.
