# Supplementary material for: Reward processing deficits: weakened self-reward association in individuals with methamphetamine addiction undergoing abstinence
Source: Front Psychol. 2025 Sep 1;16:1567735. doi: 10.3389/fpsyg.2025.1567735 (PMC12434089; doi:10.3389/fpsyg.2025.1567735)
Supplement: Supplementary file 1 [file Supplementary_file_1.docx]

# **Appendix**

**Table 1: IOS Scale**

**Instructions:** Below is a diagram depicting 7 pairs of overlapping circles with varying degrees of overlap. The circle labeled "Self" represents you, while the circle labeled "Other" represents another person. Please write the name of an acquaintance in the third type of circle relationship ( ).

**Criteria:**

- This acquaintance should only be someone with whom you exchange greetings.
- Your relationship is not very close; you are merely acquaintances who nod to each other.
- The gender of this person should match yours, and the number of characters in their name should be the same as in your own name.


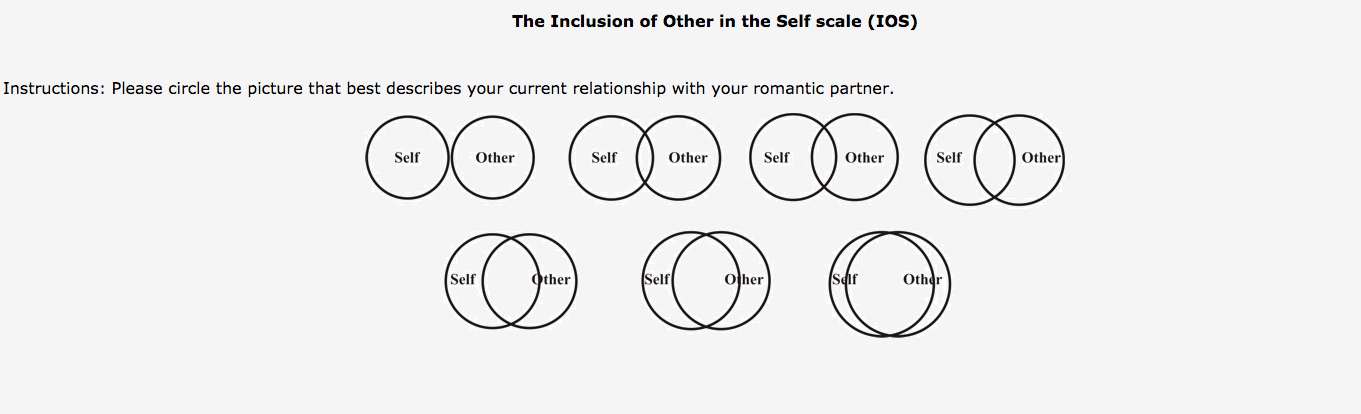


**Example Responses** (for clarity and guidance)：

If your name is "Lily" (four letters), you might write "Anna" or "Liam" for a gender-matching acquaintance.

**Table 2: Demographic Information, SCID, and DSM-V Assessment**

**Interview Questionnaire**

Interview Date:_______________ Review Date:_________________

Assessor: ________________ Reviewer:________________

**Demographic Information**

1. Participant ID: _______________

2. Date of Birth: ________________ Age: _______________

3. Gender:

(1) Male

(2) Female

4. Marital Status:

(1) Single

(2) Married

(3) Divorced

(4) Widowed

5. Education Level (Years): _______________

**Health History**

6. Have you ever sustained a brain injury (e.g., from accidents, fights, falls, drowning, gas poisoning)?

(1) Yes

(2) No

If yes, did you lose consciousness or experience a coma for more than 30 minutes?

(1) Yes

(2) No

7. Have you ever been diagnosed with a mental disorder or neurological disorder?

(1) Yes

(2) No

8. Have you taken any medication in the past week?

(1) Yes

(2) No

If yes, does this medication alter your mental state (e.g., excitement, anxiety, relaxation, drowsiness)?

___________________________________________________________________

9. Do you have any vision problems?

(1) Myopia

(4) No Issues

(2) Presbyopia

(5) Other:_________

(3) Color Blindness

If you have myopia or presbyopia, is it corrected (e.g., with glasses)?

___________________________________________________________________

10. Do you have any hearing problems?

(1) Hearing Loss

(2) Tinnitus

(3) No Issues

(4) Other: _______________

**Substance Use History**

11. When did you enter this rehabilitation center?

Year: _____ Month: _____ Day: _____

When were you arrested?

Year: _____ Month: _____ Day: _____

12. Substance Use Information:

**Indicate the first use date, last use date, average weekly usage frequency ( ___ times/week), dosage ( ___ grams/week or per use), withdrawal status, etc.**

(1) Methamphetamine (Route of Administration):

A) Oral

B) Nasal

C) Smoked

D) Cigarette

E) Non-intravenous Injection

F) Intravenous Injection

**Methamphetamine Use Questions**

**F1.** Have you ever used methamphetamine? (1) No (Skip to G1) (2) Yes

**A**. How many times have you used methamphetamine in your lifetime?____ times

1. If unknown, ask: Have you used it more than 11 times? (1) No (2) Yes

**B**. How old were you the last time you used methamphetamine? ______ years old

Last Use Timing:

(1) Within the last two weeks

(2) Two weeks to one month

(3) One month to six months

(4) Six months to one year

(5) Over one year

(U) Unknown

**C**. How many times have you used methamphetamine in the past twelve months?____ times

If unclear, ask C1. Otherwise, skip to **D**.

1. Have you used methamphetamine at least 11 times in the past twelve months?

(1) No

(2) Yes

**D**. Have you used methamphetamine at least once a week for a month or longer?

(1) No (Skip to F2)

(2) Yes

1. How old were you when you first used methamphetamine at least once a week for a month? _______________ years old

2. Last time you used methamphetamine at least once a week for a month, how old were you?_______________ years old

**F2.** How old were you when you first used methamphetamine? __________ years old

Start Time:

(1) Within the last two weeks

(2) Two weeks to one month

(3) One month to six months

(4) Six months to one year

(5) Over one year

(U) Unknow

**If at age 15 or older, skip to boxF3; otherwise, continueanswering.**

1. Before age 15, did you use methamphetamine more than once? (1) No (2) Yes

**BoxF3：**If **F1A < 11** or **F1A1 = 1**, jump to **13.**

**F3.** Have you used methamphetamine daily or almost daily? (1) No (Skip to F3B) (2) Yes

**A.** How long did you use methamphetamine almost daily? ______________ days/weeks/months/years

**B.** During your most frequent use period, how many days per month did you use methamphetamine? _______________ days

**C.** During that period, what was the average daily expenditure on methamphetamine?_______________ yuan

1. Average daily use (powder form): _______________ grams

2. Average daily use (crystal form):_______________ grams

**D.** How old were you when you started using methamphetamine most frequently? _______________ years old

**E.** How long did that period last? ________ months (if less than one month, write <1).

**F.** When you first started using methamphetamine, did you notice you were more energetic or that the effects lasted longer than for others using the same dose?

(1) No

(2) Yes

**G.** When the effects of methamphetamine wore off, did you use alcohol or other drugs to feel better?

(1) No

(2) Only alcohol

(3) Other drugs besides alcohol

If using drugs, specify:

Drug 1: _______________

Drug 2: _______________

Drug 3: _______________

Other: _______________

**H.** Have you ever injected methamphetamine? (1) No (Skip to 13) (2) Yes

1. How many times? _______________ times

2. When was the first time?

(1) Within the last two weeks

(2) Two weeks to one month

(3) One month to six months

(4) Six months to one year

(5) Over one year

(U) Unknown

3. When was the last time?

(1) Within the last two weeks

(2) Two weeks to one month

(3) One month to six months

(4) Six months to one year

(5) Over one year

(U) Unknown

**I.** Have you ever shared needles?

1. How many times? _______________ times

2. When was the first time?

(1) Within the last two weeks

(2) Two weeks to one month

(3) One month to six months

(4) Six months to one year

(5) Over one year

(U) Unknown

3. When was the last time?

(1) Within the last two weeks (4) Six months to one year

(2) Two weeks to one month (5) Over one year

(3) One month to six months (U) Unknow

**Substance Use Timeline**

1. Methamphetamine Use by Year:

| **95** | | **96** | | **97** | | **98** | | **99** | | **00** | | **01** | | **02** | | **03** | | **04** | | **05** | | **06** | | **07** | | **08** | | **09** | | **10** | | **11** | | **12** | | **13** | | **14** | | **15** | | **16** | | **17** | |
| --- | --- | --- | --- | --- | --- | --- | --- | --- | --- | --- | --- | --- | --- | --- | --- | --- | --- | --- | --- | --- | --- | --- | --- | --- | --- | --- | --- | --- | --- | --- | --- | --- | --- | --- | --- | --- | --- | --- | --- | --- | --- | --- | --- | --- | --- |
|  |  |  |  |  |  |  |  |  |  |  |  |  |  |  |  |  |  |  |  |  |  |  |  |  |  |  |  |  |  |  |  |  |  |  |  |  |  |  |  |  |  |  |  |  |  |

1. Ecstasy (MDMA) Use by Year:

| **95** | | **96** | | **97** | | **98** | | **99** | | **00** | | **01** | | **02** | | **03** | | **04** | | **05** | | **06** | | **07** | | **08** | | **09** | | **10** | | **11** | | **12** | | **13** | | **14** | | **15** | | **16** | | **17** | |
| --- | --- | --- | --- | --- | --- | --- | --- | --- | --- | --- | --- | --- | --- | --- | --- | --- | --- | --- | --- | --- | --- | --- | --- | --- | --- | --- | --- | --- | --- | --- | --- | --- | --- | --- | --- | --- | --- | --- | --- | --- | --- | --- | --- | --- | --- |
|  |  |  |  |  |  |  |  |  |  |  |  |  |  |  |  |  |  |  |  |  |  |  |  |  |  |  |  |  |  |  |  |  |  |  |  |  |  |  |  |  |  |  |  |  |  |

1. Other Substances Used by Year:

| **95** | | **96** | | **97** | | **98** | | **99** | | **00** | | **01** | | **02** | | **03** | | **04** | | **05** | | **06** | | **07** | | **08** | | **09** | | **10** | | **11** | | **12** | | **13** | | **14** | | **15** | | **16** | | **17** | |
| --- | --- | --- | --- | --- | --- | --- | --- | --- | --- | --- | --- | --- | --- | --- | --- | --- | --- | --- | --- | --- | --- | --- | --- | --- | --- | --- | --- | --- | --- | --- | --- | --- | --- | --- | --- | --- | --- | --- | --- | --- | --- | --- | --- | --- | --- |
|  |  |  |  |  |  |  |  |  |  |  |  |  |  |  |  |  |  |  |  |  |  |  |  |  |  |  |  |  |  |  |  |  |  |  |  |  |  |  |  |  |  |  |  |  |  |

1. Other__________; usage count (1) <5 times; (2) 5~10 times; (3)>10 times

**Alcohol and Tobacco Use**

13. Average Monthly Alcohol Consumption:

(1) No Alcohol

(2) Beer: _____ bottles

(3) Spirits: _____ liang (Degree: ______)

14. Average Daily Cigarette Consumption: __________ cigarettes

**Drug Use Context**

15. Where do you most commonly use methamphetamine? (Select up to 3, ranked)

(1) Bedroom ( )

(2) Living Room ( )

(3) Kitchen ( )

(4) Hotel ( )

(5) Car ( )

(6) Other: _______________ ( )

16. What situation do you find yourself in most often while using methamphetamine? (Select all that apply, indicate percentage)

(1) Alone ( %)

(2) With one friend ( %)

(3) With multiple friends ( %)

(4) Other: _______________ ( %)

17. What factors most trigger your use of methamphetamine? (Select up to 3, ranked)

(1) Methamphetamine and paraphernalia ( )

(2) Peers who use ( )

(3) Environment ( )

(4) Other: _______________ ( )

**Table 3: SCID Assessment**

?: There is insufficient information to rate as 1, 2, or 3. (Cannot recall the situation; suspicious, such as denial of complaints, but observable phenomena are present). If sufficient information is obtained later to make a judgment, it should not be rated as such.

1: Lack of evidence (clearly does not exist) or negative (clearly does not meet the criteria)

2: Sub-threshold status (almost meets the criteria, but there is still a significant gap to full compliance)

3: Meets the criteria or confirmed presence (meets or exceeds the criteria; definitely exists)

| 1. Have you ever drunk a lot of alcohol in your life? (Beer, wine, or liquor?) | 1 2 3 |
| --- | --- |
| 1. Have you ever used drugs? | 1 2 3 |
| 1. Have you ever been addicted to or taken prescription drugs in excess? | 1 2 3 |
| 1. Have you ever had a sudden feeling of fear, or anxiety, or a sudden onset of many physical symptoms of a panic attack? | 1 2 3 |
| 1. Have you ever been afraid to go out alone, stay in crowded places, wait in line, or travel by car or train? | 1 2 3 |
| 1. Do you feel afraid or uncomfortable when doing certain things in front of others, such as speaking, eating, or writing? | 1 2 3 |
| 1. Do you have any other specific fears, such as flying, seeing blood, shooting, heights, confined spaces, a certain animal or insect? | 1 2 3 |
| 1. Have you ever been troubled by thoughts that seem meaningless, thoughts that you don't want to think about, yet they keep coming back? | 1 2 3 |
| 1. Have you ever found yourself compelled to repeat an action over and over again, such as repeatedly washing hands, counting, or checking something repeatedly to ensure you've done it right? | 1 2 3 |
| 1. Have you been particularly tense or anxious in the past six months? | 1 2 3 |
| 1. Has there ever been a time when others thought you were too thin? | 1 2 3 |
| 1. Do you often lose control over your eating? | 1 2 3 |

**Table 4: Methamphetamine use disorder**

Use of methamphetamine results in clinically significant impairment or distress, meeting at least two of the following criteria within the last 12 months.

| 1. **Have you ever consumed more methamphetamine than you intended, or for a longer time than planned? (Frequently)**   Have there been times when you planned to use a certain amount, but ended up using more? For example, if you intended to use a certain amount but used it all at once instead of spreading it out over two uses. If you select "sometimes" or "no," skip to: Or did you plan to spend only a little time using it, but ended up spending much longer? How often do these situations occur?  **[SD:F10]** | □Always  □Sometimes  □Never |
| --- | --- |
| 1. **Have you had a long-standing desire to reduce or control your methamphetamine use, or have you ever tried but failed?**   Have you ever tried to completely stop using or intentionally reduce your use? Did you ultimately fail? How many times has this happened? If you select "sometimes" or "no," skip to: If you have never made a real effort to try, have you ever thought about quitting or reducing your use? How often do these thoughts occur? Is this something that has been a constant struggle for you?  **[SD: Three times or more, select frequently F9]** | □Always  □Sometimes  □Never |
| 1. **Do you spend a lot of time obtaining methamphetamine, using it, or recovering from its effects?**   Is it easy to get? Have you ever had trouble contacting your supplier and used various methods to obtain it? How much time do you spend on this (including contacting, waiting, picking up, etc.)? If you select "sometimes" or "no," skip to: When you want to use, do you set aside a specific amount of time for it? How long is that time? If you select "sometimes" or "no," skip to: How long does it take for the effects to wear off and return to normal? (Does it take several hours?)  **[SD: Have you ever spent most of your time for a month or longer using methamphetamine, obtaining it, or recovering from its effects? F6]** | □Always  □Sometimes  □Never |
| 1. **Do you experience cravings, a strong desire, or urgency to use methamphetamine?**   Do you sometimes feel a strong urge to use? If you select "sometimes" or "no," skip to: Do you ever feel an overwhelming need to use? How often does this happen?  **[SD: Have you ever had such a strong craving for methamphetamine that it was hard to think about anything else?]** | □Always  □Sometimes  □Never |
| 1. **Does frequent use of methamphetamine lead to an inability to fulfill important responsibilities at work, school, or home?**   Has your use ever interfered with your responsibilities, such as work, school, or family life? Do these situations occur often?  **[SD: 1. Have you felt depressed or uninterested in things for more than 24 hours, affecting your daily functioning? 2. Have you had difficulty concentrating or thinking clearly for more than 24 hours, affecting your daily functioning? F8]** | □Always  □Sometimes  □Never |
| 1. **Despite ongoing social or interpersonal problems caused by methamphetamine use, do you continue to use?**   Has your relationship with friends, family, or colleagues been negatively affected by your use of methamphetamine? For example, have they distanced themselves from you upon learning about your use, or have your mood swings, paranoia, or obsession created issues? After these situations arise or worsen, do you still continue to use methamphetamine? How often does this happen, knowing it causes the aforementioned problems?  **[SD: 3. Have you been overly suspicious of others for more than 24 hours, affecting your relationships? F8]** | □Always  □Sometimes  □Never |
| 1. **Have you given up or reduced important social, occupational, or recreational activities due to methamphetamine use?**   Have you abandoned or reduced important social interactions, work obligations, or hobbies after frequently using methamphetamine? Is this reduction significant?  **[SD: A. Have the above situations occurred three times or more, or lasted a month or longer? F17 6. Have you reduced contact with friends or family? F8]** | □Always  □Sometimes  □Never |
| 1. **Do you continue to use methamphetamine in situations where it poses a risk to your safety (e.g., driving, operating machinery, working at heights)?**   Do you use methamphetamine while engaging in potentially dangerous activities, such as driving, operating machinery, or working at heights? How often does this happen?  **[SD: Have you driven or operated dangerous equipment while under the influence, or have you ever sustained injuries from methamphetamine use, such as severe falls, cuts, burns, or injuries from accidents? F13]** | □Always  □Sometimes  □Never |
| 1. **Despite recognizing that methamphetamine use causes persistent or recurring physical or psychological issues, do you continue to use?**   Have you ever experienced psychological issues such as depression, irritability, or paranoia due to methamphetamine use? If you select "sometimes" or "no," skip to: Has methamphetamine use ever caused significant physical problems or worsened existing issues? Even knowing these problems exist, do you still use methamphetamine? How often does this occur?  **[SD: Has methamphetamine use ever required you to seek treatment due to overdose, or has it led to other serious health problems? Do you continue to use despite knowing it affects your health? F18]** | □Always  □Sometimes  □Never |
| 1. **Tolerance: Defined as either of the following:**   a. **A noticeable increase in the amount of methamphetamine needed to achieve intoxication or the desired effect.**  How much did you initially use? Over time, have you needed to use more to achieve the desired effects? Is this change significant? If you select "sometimes" or "no," skip to:  b. **Continuing to use the same amount results in significantly reduced effects.**  If you use the same amount as before, does the effect feel the same or worse than before? Is this difference significant?  **[SD: If you have increased your usual amount by 50% or more, please answer yes. F11]** | □Always  □Sometimes  □Never |
| 1. **Withdrawal Symptoms: Defined as either of the following:**   a. **Typical withdrawal symptoms from methamphetamine.**  After using for a period (weeks or longer), if you reduce your intake or suddenly stop, what changes do you experience? (Negative mood and two or more of the following physiological changes: fatigue, vivid unpleasant dreams, insomnia or hypersomnia, increased appetite, psychomotor agitation or retardation). Are these changes significant? If you select "sometimes" or "no," skip to:  b. **Using methamphetamine or similar substances alleviates or avoids withdrawal symptoms.**  After experiencing the above changes, does using methamphetamine help alleviate these symptoms? Is this relief significant?  **[SD: Have you used methamphetamine three times or more to avoid the above symptoms? Select frequently F12]** | □Always  □Sometimes  □Never |

**Indicate Current Severity Level**  **Mild:** Presence of 2-3 symptoms □

**Moderate:** Presence of 4-5 symptoms □

**Severe:** Presence of 6 or more symptoms □

*Note: Only if "frequently" is selected for Question 1 will it count as having symptoms. For all other questions, selecting "sometimes" or "frequently" will count as having symptoms.*

**Indicate**

**Early Remission:** Previously met the criteria for methamphetamine use disorder, but in the past 3 to 12 months, none of the criteria have been met (excluding Criterion 4) □

**Sustained Remission:** Previously met the criteria for methamphetamine use disorder, but in the past 12 months, none of the criteria have been met (excluding Criterion 4) □

**Indicate**

**In a Controlled Environment** □
